# Probing the structure and function of the protease domain of botulinum neurotoxins using single-domain antibodies

**DOI:** 10.1371/journal.ppat.1010169

**Published:** 2022-01-06

**Authors:** Kwok-ho Lam, Jacqueline M. Tremblay, Kay Perry, Konstantin Ichtchenko, Charles B. Shoemaker, Rongsheng Jin

**Affiliations:** 1 Department of Physiology and Biophysics, University of California, Irvine, California, United States of America; 2 Tufts Cummings School of Veterinary Medicine, North Grafton, Massachusetts, United States of America; 3 NE-CAT, Department of Chemistry and Chemical Biology, Cornell University, Argonne National Laboratory, Argonne, Illinois, United States of America; 4 Department of Biochemistry and Molecular Pharmacology, New York University Grossman School of Medicine, New York, New York, United States of America; University of Pittsburgh School of Medicine, UNITED STATES

## Abstract

Botulinum neurotoxins (BoNTs) are among the deadliest of bacterial toxins. BoNT serotype A and B in particular pose the most serious threat to humans because of their high potency and persistence. To date, there is no effective treatment for late post-exposure therapy of botulism patients. Here, we aim to develop single-domain variable heavy-chain (VHH) antibodies targeting the protease domains (also known as the light chain, LC) of BoNT/A and BoNT/B as antidotes for post-intoxication treatments. Using a combination of X-ray crystallography and biochemical assays, we investigated the structures and inhibition mechanisms of a dozen unique VHHs that recognize four and three non-overlapping epitopes on the LC of BoNT/A and BoNT/B, respectively. We show that the VHHs that inhibit the LC activity occupy the extended substrate-recognition exosites or the cleavage pocket of LC/A or LC/B and thus block substrate binding. Notably, we identified several VHHs that recognize highly conserved epitopes across BoNT/A or BoNT/B subtypes, suggesting that these VHHs exhibit broad subtype efficacy. Further, we identify two novel conformations of the full-length LC/A, that could aid future development of inhibitors against BoNT/A. Our studies lay the foundation for structure-based engineering of protein- or peptide-based BoNT inhibitors with enhanced potencies and cross-subtypes properties.

## Author summary

Botulinum neurotoxins (BoNTs) are extremely toxic to humans by causing flaccid paralysis of botulism. The catalytic light chain (LC) of BoNTs is the warhead of the toxin, which is mainly responsible for BoNT’s neurotoxic effects. As an endopeptidase, LC is delivered by the toxin to inside neurons where it specifically cleaves neuronal SNARE proteins and causes muscle paralysis. While the currently available equine and human antitoxin sera can prevent further intoxication, they do not promote recovery from paralysis that has already occurred. We strike to develop single-domain variable heavy-chain (VHH) antibodies targeting the LC of BoNT/A (LC/A) and BoNT/B (LC/B) as antidotes to inhibit or eliminate the intraneuronal LC protease. Here, we report the identification and characterization of large panels of new and unique VHHs that bind to LC/A or LC/B. Using a combination of X-ray crystallography and biochemical assays, we reveal that VHHs exploit diverse mechanisms to interact with LC/A and LC/B and inhibit their protease activity, and such knowledge can be harnessed to predict their specificity towards different toxin subtypes within each serotype. We anticipate that the new VHHs and their characterization reported here will contribute to the development of improved botulism therapeutics having high potencies and broad specificities.

## Introduction

Botulinum neurotoxins (BoNTs) are extremely potent toxins to humans and cause flaccid paralysis. These toxins are widely distributed in nature and relatively simple to produce in quantity. For these reasons, BoNTs are listed by the Centers of Disease Control and Prevention (CDC) as Tier 1 Select Agents. BoNTs also are highly variable in nature with at least seven major serotypes reported (A-G), most of which forms a group with multiple variant subtypes. This extreme natural variability seriously complicates the development of botulism treatments. Currently the only available therapeutics for botulism are equine or human antitoxin serum products which are administered intravenously to patients exposed to toxin. Efforts are well underway to develop monoclonal antibody (mAb) antitoxins with the goal to replace current antiserum products [1–5]. While antitoxin treatments prevent further intoxication, they do not promote recovery from paralysis that has already occurred.

Several groups are working to develop the next generation of botulism antidotes with the goal to inhibit or eliminate the intraneuronal protease that causes paralysis. Our group seeks to develop single-domain antibodies (sdAbs) to be employed as components of biomolecular antidotes that target the various BoNT proteases. These sdAbs are derived from the heavy-chain-only antibodies produced by camelid animals. The heavy chain variable regions of these antibodies, called VHHs or nanobodies, are small sdAbs of ~14 kDa that are tightly folded, stable, and highly amenable to recombinant expression and engineering as multimers or as fusions to other vehicles. VHHs also have a preference for binding to conformational epitopes that often are functional sites on proteins such as enzyme active sites or receptor-binding sites. Multimers of toxin-neutralizing VHHs have been employed in the generation of botulism antitoxins and shown to have substantially improved potencies [6,7], particularly when the multimer designs are guided by structural information [8].

All BoNTs have a catalytic light chain (LC), which is a Zn^2+^-endopeptidase that specifically cleaves neuronal SNARE proteins and is mainly responsible for BoNT’s neurotoxic effects, while the heavy chain (HC) mediates toxin attachment to neurons and delivers the LC into the cytosol. Two recent reports demonstrated that animals with symptomatic botulism can be rescued by treatments with biomolecular antidotes consisting of an atoxic BoNT delivery vehicle fused to VHHs that bind and inhibit the LC of the intoxicating BoNT [9,10]. These findings suggest that more effective botulism antidotes will be possible with the identification of VHHs possessing higher potency to inhibit the LC of BoNT, particularly those that are broadly active on most or all natural subtypes of each BoNT serotype.

In this study, we report the identification of large panels of new and unique VHHs that bind to LC/A or LC/B, many having potent activities to inhibit the protease function. To gain insight into the mechanisms of neutralization, structural studies were performed to identify the protease binding sites for many of these VHHs. The structural information also reveals the potential of these VHHs to bind and neutralize other known natural subtypes of these two proteases. We anticipate that the new VHHs and their characterization reported here will contribute to the development of improved botulism therapeutics having higher potencies and broader specificities than those reported to date.

## Results

### Discovery and characterization of new VHHs targeting the LC of BoNT/A and BoNT/B

Prior reports have described the characterization of several VHHs targeting BoNT/A and BoNT/B [6,8], some of which recognize the light chain protease domains of these toxins (LC/A and LC/B). VHHs have also been identified specifically for their binding to LC/A [11,12]. These VHHs were typically selected based on their ability to bind to the LC in the context of holotoxins or the isolated LC, which were immobilized by coating to plastic plates. New information, particularly with the LC of BoNT/E (LC/E), has demonstrated that BoNT LC can become conformationally altered when coated to plastic plates [7]. VHHs are known to be highly dependent on 3D conformation of their respective epitopes for binding [13] and we speculated that many useful VHHs were likely missed from earlier discovery efforts in which VHHs were selected for their affinity to plastic-coated BoNT proteases. We thus performed new discovery efforts for VHHs binding LC/A and LC/B in which we employed protease targets that were antibody-captured so as to retain their native conformational state in solution.

To discover new VHHs to LC/A, we prepared a new phage-displayed library from two alpacas hyperimmunized with LC/A and selected VHHs that bound to LC/A immobilized by ALc-B8 VHH [12]. ALc-B8 was previously shown to bind soluble LC/A with high affinity and inhibit its protease activity. The VHH capture panning resulted in the discovery of 13 new unique LC/A-binding VHHs that had not been previously identified by panning on plastic-coated LC/A [12]. The sequences of these new VHHs (JPU series) are shown in S1 Fig. Also included for comparison are sequences of several previously identified LC/A-binding VHHs that were reported elsewhere (ciA-D1, ciA-D12, ciA-F12, ciA-H7, ALc-H7) [6,12].

A panel of new LC/B-binding VHHs was identified from a phage-displayed library prepared from two alpacas immunized with purified LC/B and selected for binding to LC/B captured to plastic plates with VHH BLc-B10 or JND-E4. VHH BLc-B10 was previously identified by panning on plastic-coated LC/B [12], which was used here as a LC/B-capturing agent to discover seven unique new LC/B-binding VHHs (JND-series). One of the new VHHs, JND-E4, was subsequently employed as the LC/B-capturing VHH in a new round of panning in order to block a dominant epitope, which led to the discovery of an additional seven unique LC/B-binding VHHs (JSG series). The sequences of these 14 new, unique VHHs are shown in S2 Fig aligned with the sequences of BLc-B10 and three other previously reported LC/B-binding VHHs (JLJ-F9, JLJ-G3, JNE-B10) discovered following selection on the catalytically inactive BoNT/B holotoxin (ciBoNT/B) [8].

These newly identified LC/A- and LC/B-binding VHHs were characterized for their target binding properties and their potency to inhibit the target proteases (Tables 1 and 2). They were first tested for the apparent binding affinities to the appropriate BoNT holotoxin (ciBoNT/A or ciBoNT/B) and isolated protease domain (LC/A or LC/B). Dilution ELISAs to the LCs were performed both by coating the targets onto plastic plates or by immobilizing the soluble protease with a capture antibody. As found with the LC/E-binding VHHs [7], binding affinity was often substantially compromised by coating the proteases onto plastic plates. Even using the less denaturing tissue culture plastic plates to coat LC/A or LC/B, the binding was significantly reduced compared to the captured LCs for many of the VHHs. Most of the VHHs displayed sub-nM apparent affinities to the captured LCs. Interestingly, the majority of new VHHs selected on the isolated LCs showed poor or undetectable binding to the holotoxins. This is due to the unique structure of BoNT holotoxin, where a portion of the heavy chain wraps around the LC like a belt that is believed to act as a substrate surrogate [14]. Therefore, the epitopes of many of these newly identified LC-binding VHHs may be masked by the belt in the context of the holotoxin. Competition ELISAs were performed to provide evidence that two or more VHHs bind to overlapping or nearby epitopes on the LCs. From these ELISAs, VHHs representing six different epitope ‘competition groups’ were found on LC/A and five groups found on LC/B.

**Table 1:** A summary of anti-BoNT/A VHH characterization data.

VHH name^A^	ciBoNT/A-binding EC_50_, nM coated^B^	LC/A1-binding EC_50_, nM coated^C^	LC/A1-binding EC_50_, nM captured^D^	LC/A1 protease inhibition^E^	Competition group^F^
ALc-B8	NB	3	0.5	++	1
ALc-H7	Trace	5	0.5	++	1
ciA-D1	3	1	0.4	-	2
ciA-D12	3	1	0.2	-	3
ciA-F12	0.2	1	0.2	-	4
ciA-H7	0.2	3	0.1	-	2
JPU-A1	1	10	0.4	-	2
JPU-A5	Trace	10	0.1	++++	5
JPU-A11	Trace	10	0.4	++	6
JPU-B5	NB	Trace	0.8	-	5
JPU-B9	NB	NB	0.8	+	5
JPU-C1	NB	20	0.2	++++	2
JPU-C10	NB	Trace	0.2	++++	5
JPU-D12	NB	5	0.2	+++	6
JPU-G3	NB	5	0.2	+++	2
JPU-G7	15	10	3	-	2
JPU-G11	Trace	1	0.5	+++	1
JPU-G12	10	10	0.5	-	2
JPU-H7	3	3	0.8	-	3

^A^Lab code given to each VHH

^B^The EC_50_ was the VHH concentration that produced about 50% of the peak signal on a serial dilution ELISA on ciBoNT/A-coated Costar plates

^C^The EC_50_ was the VHH concentration that produced about 50% of the peak signal on a serial dilution ELISA on LC/A-coated Costar plates

^D^The EC_50_ was the VHH concentration that produced about 50% of the peak signal on a serial dilution ELISA on LC/A captured by a LC/A-binding VHH (value shown is the lowest found after testing with several different capture VHHs)

^E^Based on protease inhibition assays such as shown in S3 Fig and described in Materials and Methods

^F^Based on competition analysis as described in Materials and Methods

NB = no significant binding

Trace = some signal; too low for EC_50_ assessment (i.e. EC_50_ >125 nM)

- Showed no inhibition on SNAP25-cleavage assays (e.g. S3 Fig)

+ Showed weak inhibition on SNAP25-cleavage assays (e.g. S3 Fig)

++ Showed moderate inhibition on SNAP25-cleavage assays (e.g. S3 Fig)

+++ Showed strong inhibition on SNAP25-cleavage assays (e.g. S3 Fig)

++++ Showed strong and persistent inhibition on SNAP25-cleavage assays (e.g. S3 Fig)

**Table 2:** A summary of anti-BoNT/B VHH characterization data.

VHH name^A^	ciBoNTB-binding EC_50_, nM captured^B^	LC/B1-binding EC_50_, nM coated^C^	LC/B1-binding EC_50_, nM captured^D^	LC/B1 protease inhibition^E^	Competition group^F^
BLc-B10	Trace	5*	10	-	1
JLJ-F9	0.5	5	0.8	+/-	2
JLJ-G3	0.1	10	3	+	3^#^
JLO-G7	0.1	0.5	0.5	+/-	2
JND-A12	15	2	0.5	-	2
JND-B4	0.1	0.8	0.5	+/-	2
JND-C7	Trace	3	0.8	+/-	4^#^
JND-E4	Trace	1	0.3	-	4^#^
JND-E5	Trace	5	2	-	4^#^
JND-E9	0.5	1	0.5	-	2
JND-F3	NB	10	0.8	-	4
JNE-B10	0.2	1	0.2	-	2
JSG-B8	25	0.8	0.1	-	2
JSG-B10	15	2	0.1	-	2
JSG-C1	Trace	5	0.5	+++	5
JSG-F6	Trace	Trace	2	+	5
JSG-G1	Trace	1	0.1	+++	5
JSG-G10	Trace	Trace	3	++	5
JSG-G11	NB	25	0.5	++	5

^A^Lab code given to each VHH

^B^The EC_50_ was the VHH concentration that produced about 50% of the peak signal on a serial dilution ELISA on JEQ-H11-captured ciBoNT/B

^C^The EC_50_ was the VHH concentration that produced about 50% of the peak signal on a serial dilution ELISA on LC/B-coated Costar plates

^D^The EC_50_ was the VHH concentration that produced about 50% of the peak signal on a serial dilution ELISA on LC/B captured by a LC/B-binding VHH (value shown is the lowest found after testing several different VHHs)

^E^Based on protease inhibition assays such as shown in S4 Fig and described in Materials and Methods

^F^Based on competition analysis as described in Materials and Methods

*Binds with 0.3 nM EC_50_ to LC/B coated to Nunc plastic

^#^Partially overlapping epitopes

NB = no significant binding by dilution ELISA at 125 nM

Trace = some signal; too low for EC_50_ assessment (i.e. EC_50_ >125 nM)

- Showed no inhibition on VAMP-cleavage assays (e.g. S4 Fig)

+/- Showed weak inhibition only at high concentrations on VAMP-cleavage assays

+ Showed moderate inhibition on VAMP-cleavage assays (e.g. S4 Fig)

++ Showed strong inhibition on VAMP-cleavage assays, not very persistent (e.g. S4 Fig)

+++ Showed strong and persistent inhibition on VAMP-cleavage assays (e.g. S4 Fig)

In addition to their binding properties, these VHHs were assessed for their ability to inhibit the targeted protease’s activity for their SNAP25 or VAMP2 substrates (Tables 1 and 2 and S3 and S4 Figs). In addition to the previously reported LC/A inhibitor ALc-B8, several additional VHHs displayed potent activities to inhibit the protease cleavage of SNAP25. Of interest, VHHs that inhibit LC/A were identified in four different competition groups (1, 2, 5, 6), indicating that multiple protease-inhibiting sites exist. This finding is substantially extended through structural studies shown below. It was not surprising to identify VHHs with varying potencies to inhibit protease function in the same competition group as each VHH binds its target in a unique manner and VHHs can bind outside of the substrate-binding site while being impeded in LC binding by the presence of a nearby protease-inhibiting VHH. For LC/B, many VHHs were found to inhibit VAMP2 cleavage by the protease although they were found in only two LC/B-binding competition groups (2 and 5), while all of the most potent and persistent VHHs were members of the same competition group 5. Note that VHHs inhibiting LC protease activity does not imply any ability to block extracellular intoxication of cells by the toxin.

### Structural studies on VHHs binding to LC/A or LC/B

**Fig 1:**
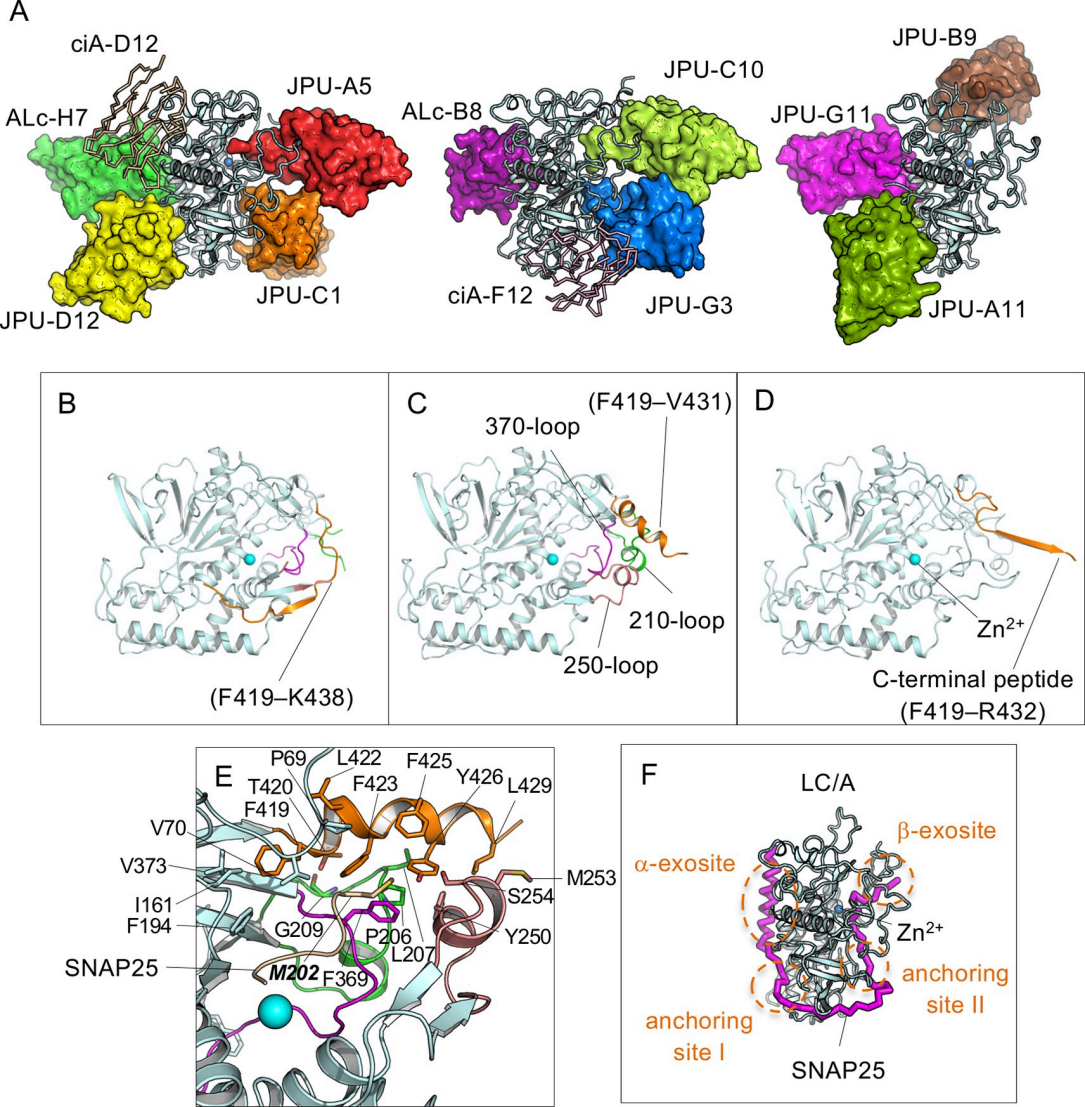
Overall structures of VHHs in complex with LC/A. (A) Structures of LC/A–VHH complexes. Inhibitor VHHs are represented in surface. Non-inhibitor VHHs (ciA-D12 and ciA-F12) are shown as ribbon. Duplicated VHH structures are omitted from the figure for clarity. (B–D) The structures of the JPU-B9-bound fLC/A (B), and the JPU-C10-bound fLC/A (C), and LC/A in BoNT/A holotoxin (PDB code 3BTA) (D). The 210-, 250-, 370-loops and C-terminal-peptide of LC/A are highlighted in green, pink, magenta, and orange, respectively. (E) Superposition of the JPU-C10-bound fLC/A with SNAP25(197–202)-bound LC/A (PDB code: 3DDA) highlighting the potential interaction between the C-terminal residues of fLC/A with P5’ M202 (shown as stick) of SNAP25. LC/A is colored as that shown in (B–D) while a short SNAP25 peptide is colored wheat. The interacting residues between the C-terminal peptide with the surrounding loops on LC/A are drawn as sticks. (F) The structure of the LC/A–SNAP25(146–204) complex (PDB ID: IXTG), where SNAP25 is shown as a purple ribbon model. The approximate regions of the α- and β-exosites and the anchoring sites I and II are indicated by dotted circles.

To investigate the inhibition mechanisms of the anti-LC/A and anti-LC/B VHHs, we selected at least two members from each of the LC/A-inhibiting VHH competition groups (groups 1, 2, 5, 6 in Table 1) and one member from each LC/B-inhibiting VHH competition group (groups 2, 3, 5 in Table 2) for further study. Inhibitory VHHs within different competition groups must each bind at unique sites associated with protease function. In total, we chose 10 anti-LC/A and 3 anti-LC/B VHHs for structural studies. Either full-length LC (residues 2–438 of LC/A, named fLC/A; 1–441 of LC/B, named fLC/B) or C-terminally truncated LC forms (residues 1–420 of LC/A, named sLC/A; 1–425 of LC/B, named sLC/B) were employed because truncated BoNT LC has been reported to promote improved protein crystallization [15]. Two of the anti-BoNT/A VHHs, JPU-C10 and JPU-B9, bound only to fLC/A and hence fLC/A was used for these two VHHs. Initially, the selected VHHs were individually co-purified with the LC for crystallization. However, this approach resulted in poorly diffracted crystals in some cases (e.g. sLC/A–JPU-A5 diffracted to ~7 Å). As an alternative, several selected non-competing VHHs were mixed together with the LC for co-crystallization since VHHs are known to promote crystal packing [16]. These included some VHHs that bind to distinct, non-inhibiting LC epitopes such as the previously reported ciA-F12 and ciA-D12 VHHs [6], in order to facilitate crystallization (Fig 1A). After extensive screening, we successfully determined the structures of sLC/A in complex with five VHHs including JPU-A5, ALc-H7, JPU-C1, JPU-D12 and ciA-F12; fLC/A in complex with six VHHs including ALc-B8, JPU-C10, JPU-G3, JPU-D12, ciA-F12 and ciA-D12; fLC/A bound to three VHHs: JPU-B9, JPU-A11, and JPU-G11; sLC/B bound to JLJ-G3 and JNE-B10; and fLC/B bound to JSG-C1, all in resolutions ranging from 1.82–2.86 Å (S1 Table).

### Structural plasticity of the C-terminus of LC/A is implicated in its enzymatic activity

The full length LC/A, fLC/A, was previously reported to be unsuitable for protein crystallization because of a tendency to aggregate and the natural flexibility of its C-terminal peptide (residues 421–438) [17]. This is why all currently available crystal structures of LC/A are based on the truncated LC/A. However, the C-terminus of fLC/A is considered functionally important as it has been reported to play roles in subcellular localization [18], persistence in neurons [19,20], and catalytic activity [21]. Therefore, it was exciting to identify JPU-C10 and JPU-B9 as VHHs that bind to fLC/A, but not to sLC/A, indicating the direct involvement of the C-terminus of LC/A in VHH binding and possibly in protease activity.

In order to better understand the function of this important C-terminal region on fLC/A, we determined the structures of fLC/A in complex with these two VHHs. Surprisingly, the C-terminal region of fLC/A adopts two different conformations in these two VHH-bound complexes, further supporting the hypothesis that this region possesses natural flexibility in solution (Fig 1B and 1C). The two different conformations of fLC/A appear to have been captured as a result of their binding to VHHs. This conformational capture is not surprising as VHHs are widely used for mechanistic studies of the dynamic conformational states of enzymes and membrane proteins [22]. In the JPU-B9-bound fLC/A, the C-terminal peptide forms a loop and a short β-strand which is similar to the conformation of LC/A observed in the context of holotoxin (Fig 1D) [23]. A major difference is that the β-strand interacts with the N-terminal region of the HC in the holotoxin while it is stabilized by pairing with the 250-loop in fLC/A. In contrast, the C-terminal peptide of the JPU-C10-bound fLC/A forms an amphipathic α-helix (F419–L429) in which the hydrophobic side is partially buried and facing the active site pocket while the hydrophilic residues are solvent-exposed (Fig 1C). We did not observe the electron density for residues R432 to K438 in the JPU-C10-bound fLC/A which are solvent-exposed and likely to have high flexibility. Therefore, the C-terminus of LC/A appears to adopt at least two distinct conformations, referred to as the β-form stabilized by JPU-B9 and the α-form captured by JPU-C10. In both conformations, residue C430 of fLC/A is solvent-exposed and thus likely available to form scrambled disulfide bonds with neighboring molecules in solution. This feature may explain why fLC/A is prone to aggregation and typically unsuitable for structural studies [24].

Prior functional studies showed that the C-terminal peptide of fLC/A contributes to catalysis of SNAP25 cleavage and substrate binding [18,21,24]. Structural analysis of fLC/A suggests two potential roles for the C-terminal peptide in its enzymatic activity. First, the C-terminal helix in the α-form may form a latch that interacts with the 210-, 260-, and 370-loops of LC/A through hydrophobic interactions and thus promotes catalysis by stabilizing the active conformation of LC/A (Fig 1C). In contrast, the 210-loop and 260-loop in the β-form are solvent exposed with high local flexibility resembling that of the apo-LC/A (Fig 1B and 1D). Second, the C-terminal helix in the α-form, together with the 210-loop and 260-loop, form a hydrophobic pocket which has clusters of aromatic residues (e.g. F423, F425, Y426) that could facilitate the docking of the C-terminal region of SNAP25 to LC/A. In fact, the α-conformation shares a similarity with the SNAP25(197–202)-bound LC/A (PDB code 3DDA) [15,25] and structural analysis shows that this hydrophobic pocket involving the C-terminal helix could facilitate the binding of the P5’ M202 residue of SNAP25 to the catalytic site (Fig 1E). Taken together, our data suggests the C-terminus of fLC/A can dynamically alter between at least two conformations, defined here as the α- and the β-forms, and that these forms, and possibly others, likely participate in the regulation of SNAP25 substrate binding and product release. As such, the structures of fLC/A should be more suitable and informative than sLC/A when performing studies of VHH inhibition mechanisms and for the development of peptide or small molecule inhibitors of LC/A.

### VHHs exploit diverse mechanisms to interact with LC/A and inhibit its protease activity

LC/A recognizes SNAP25 at the catalytic site as well as at multiple distant sites (termed exosites) in a manner such that SNAP25 effectively encircles the protease (Fig 1F) [15,25]. Indeed, this unique substrate-binding mode closely resembles the association of LC/A with the heavy chain ‘belt’ region in the context of holotoxin [15]. It is now well established that the N-terminal residues of SNAP25 are docked to a hydrophobic surface of LC/A called the α-exosite, the middle unstructured region of SNAP25 interacts with LC/A through two anchoring points called I and II, and the C-terminus of SNAP25 forms a beta-strand and interacts with the catalytic β-exosite of LC/A (Fig 1F) [15,25].

Comparing the structures of SNAP25-bound LC/A with the structures of LC/A in complex with protease-inhibiting VHHs reveals diverse mechanisms by which these VHHs inhibit LC/A cleavage by competing with SNAP25 binding at different sites. Specifically, strong LC/A protease inhibiting VHHs could be classified into four groups: (1) three VHHs bind to the α-exosite of LC/A (ALc-B8, JPU-G11 and ALc-H7); (2) two bind to anchoring site I (JPU-A11 and JPU-D12); (3) two bind anchoring site II (JPU-C1 and JPU-G3); and (4) two bind the β-exosite of LC/A (JPU-A5 and JPU-C10). Consistent with our competition studies, VHHs in each of these four groups belong to the same competition group and bind to overlapping epitopes (Table 1). Structures were also obtained for three non-inhibitory VHHs in complex with LC/A, and in each case, as expected, the structures suggest these VHHs should not interfere with SNAP25 binding to the protease. The inhibition mechanisms underlying these four groups of VHHs are discussed in details as follows.

#### VHHs targeting the β-exosite of LC/A

**Fig 2:**
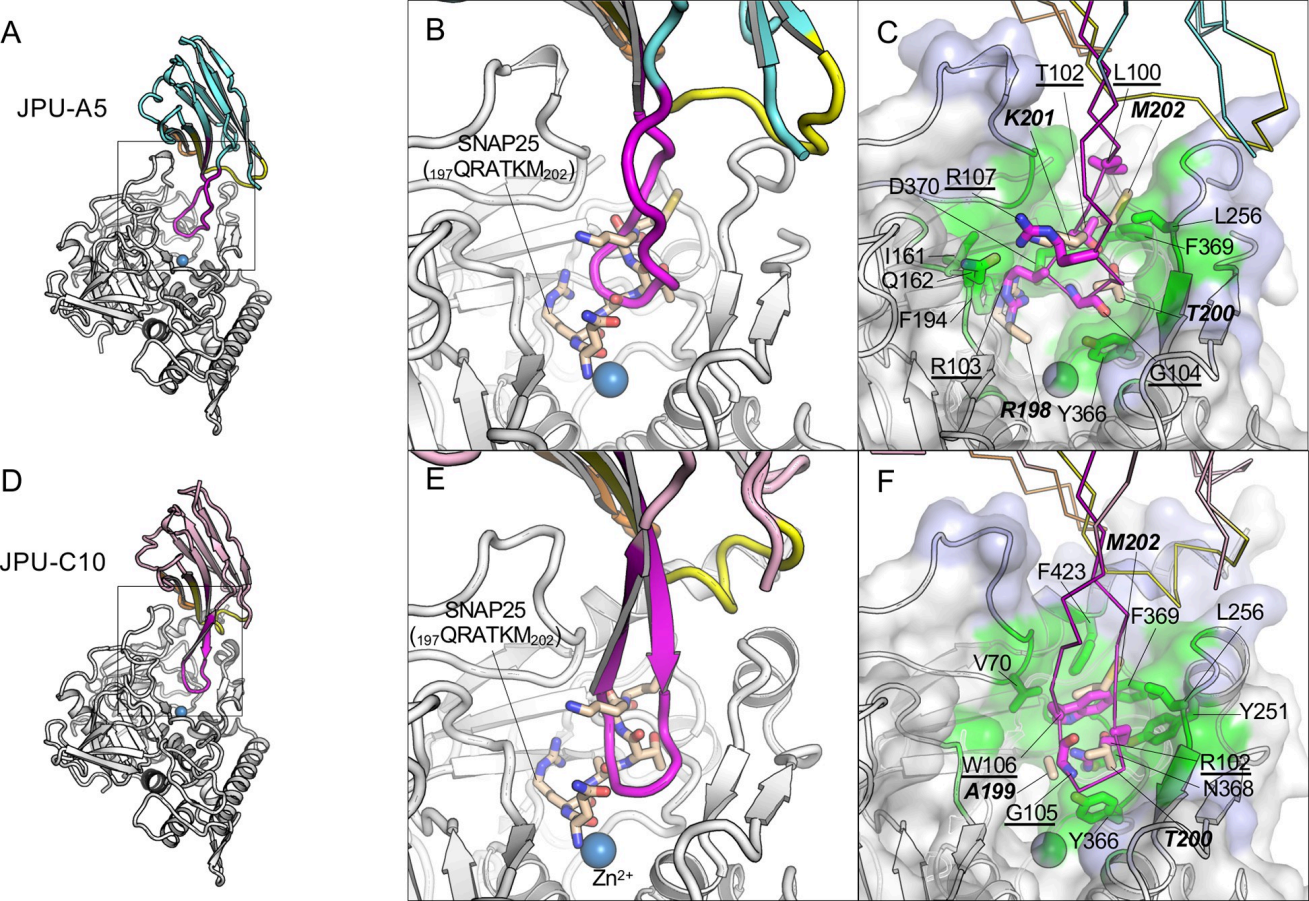
JPU-A5 and JPU-C10 inhibit the binding of SNAP25 to the β-exosite of LC/A. (A, D) Cartoon representation of JPU-A5 (A) and JPU-C10 (D) in complex with LC/A. The CDR1, 2, and 3 are colored yellow, orange, and magenta, respectively. The framework region (FR) of JPU-A5 and JPU-C10 are colored cyan, pink, and light green, respectively. (B, E) Superposition of LC/A–JPU-A5 (B) or LC/A–JPU-C10 (E) with the SNAP25(197–202)-bound LC/A (PDB ID: 3DDA). Only the VHH-bound LC/A colored white is represented in (B) and (E) for clarity, while SNAP-25 peptide is colored wheat. (C, F) JPU-A5 (C) and JPU-C10 (F) competes with SNAP25 residues for LC/A binding. Molecular surface of LC/A is presented to highlight the binding epitopes. The VHH-binding epitopes on LC/A are colored blue, while the regions overlapping with SNAP25 binding are colored green. Key VHH residues that interfere with SNAP25 binding and the corresponding interacting LC/A residues are drawn as sticks.

Two potent LC/A inhibitors, JPU-A5 and JPU-C10, bind to the deep active site pocket present at the LC/A β-exosite where the cleavage site and the C-terminus of SNAP25 become associated (Fig 2). JPU-C10 buried a surface area (BSA) of ~1215 Å^2^ on LC/A (calculated by PDBePISA v1.52) with a surface complementarity (SC) score of 0.731 [26] which is higher than the average SC value of ~0.7 for antibody-antigen complexes [27]. JPU-A5 binding buried a BSA of ~1033 Å^2^ with an SC score of 0.686 (S2 Table). Both of the VHHs interact with the 60-, 250-, and 370-loops of LC/A known to be crucial for enzymatic catalysis (Fig 2A–2F). The JPU-A5 or JPU-C10–bound LC/A adopted a conformation closely resembling that of LC/A bound to a short peptide of SNAP25(197–202) (r.m.s.d. = 0.49 Å), which represents a pre-cleavage intermediate conformation of LC/A and defines how SNAP25 enters the cleavage site pocket and the S1’–S5’ subsites of LC/A [25]. Interestingly, the CDR3 of both JPU-A5 and JPU-C10 project deeply into the active site of LC/A (Fig 2B and 2E). However, since these two VHHs have completely different sequences in their CDR3, they exploit distinct ways to occupy the S1’–S5’ subsites of LC/A to achieve inhibition.

For JPU-A5, its R103 inserts into the S1’ pocket of LC/A by forming a salt-bridge with D370, a hydrogen bond with the carbonyl group of I161, and a cation-π interaction with F194, which is similar to how R198 of SNAP25 engages the S1’ subsite on LC/A (Fig 2C) [25]. Interestingly several peptide inhibitors of LC/A also exploit the S1’ subsite of LC/A using arginine residues to achieve high affinity binding [28–30]. Besides R103, G104 and R107 in CDR3 of JPU-A5 form H-bonds with S3’ and S4’ subsite residues Y366 and Q162 of LC/A, respectively; T102 and L100 in CRD3 interact with the S5’ subsite residues F369 and L256 of LC/A. Furthermore, CDR1 and CDR2 of JPU-A5 also participate in the complex formation by interacting with the 60- and 250-loop of LC/A through hydrogen bonds.

JPU-C10 does not occupy the S1’ subsite of LC/A. Rather, its R102 and G105 interact with S2’ and S3’ subsite residues N368, Y251, and Y366 of LC/A (Fig 2F). Additionally, its W106 forms hydrophobic interaction with the S5’ subsite residues Y251, L256, F369, V70, and F423 of LC/A. The CDR3 of JPU-C10 is so close to the cleavage site that its binding should impose steric hindrance that occludes SNAP25 access to the cleavage site pocket. JPU-C10 not only interacts extensively with the S1’–S5’ sites through its CDR3, it also binds to the C-terminus of LC/A that forms a helix in the complex. In summary, both JPU-A5 and JPU-C10 potently inhibit LC/A activity by blocking SNAP25 interaction with the cleavage pocket of LC/A.

#### VHHs targeting the anchoring site II

**Fig 3:**
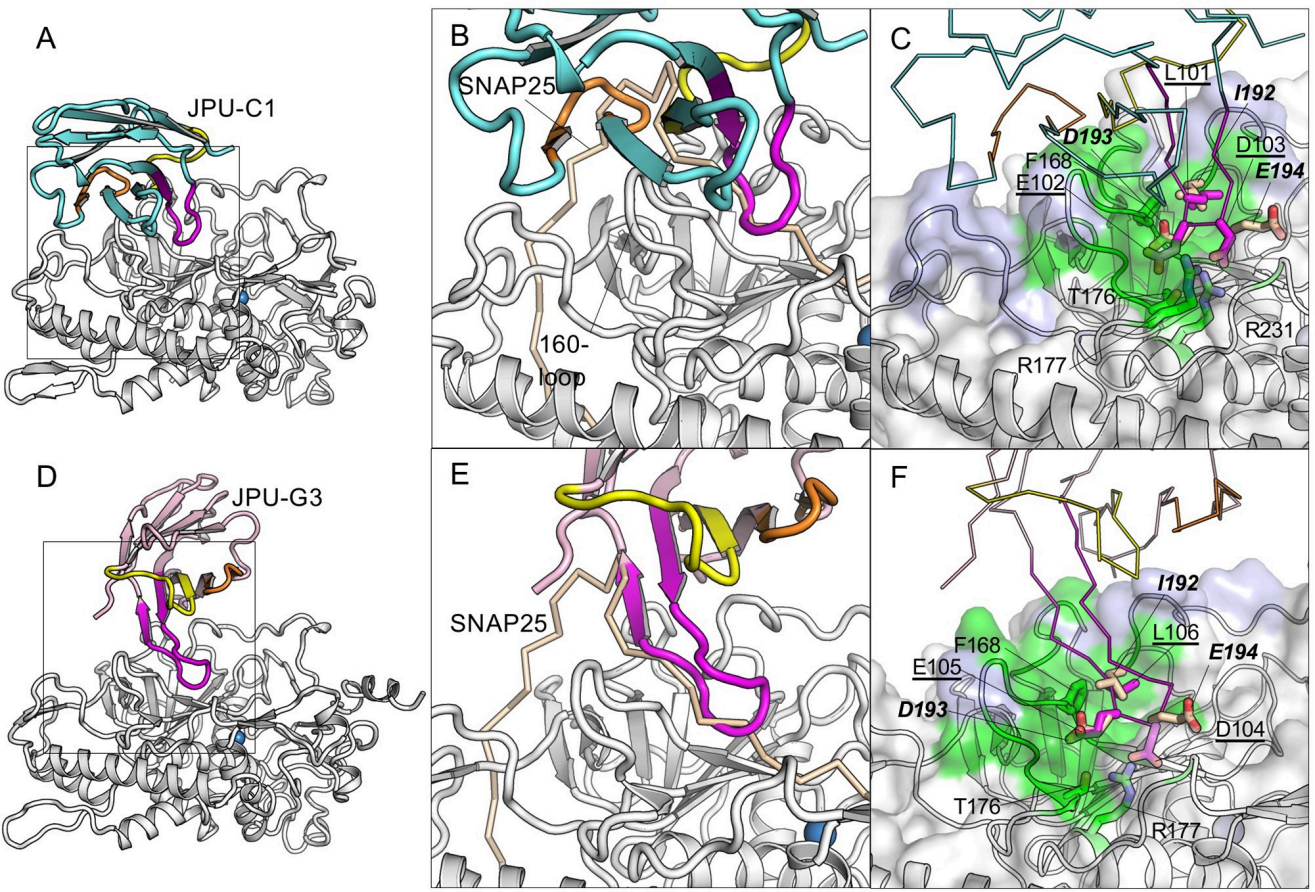
JPU-C1 and JPU-G3 block the interaction of SNAP25 to the anchoring site II of LC/A. (A, D) Cartoon representation of JPU-C1 (A) or JPU-G3 (D) in complex with LC/A. The framework region of JPU-C1 and JPU-G3 are colored cyan and pink respectively. The CDR1, 2, and 3 are colored yellow, orange, and magenta, respectively. (B, E) Superposition of LC/A–JPU-C1 (B) or LC/A–JPU-G3 (E) with the SNAP25(146–204)-bound LC/A (PDB ID: 1XTG). Only the VHH-bound LC/A is represented. LC/A and the long SNAP25 peptide are colored white and wheat, respectively. (C, F) The VHH residues competing with SNAP25 residues for LC/A binding are drawn as sticks. The epitopes are colored as that shown in Fig 2.

VHHs JPU-C1 and JPU-G3 both bind to the anchoring site II of LC/A and inhibit LC/A protease (Table 1 and S3 Fig). JPU-C1 interacts extensively with LC/A involving all three CDRs, displaying a large BSA of 1010 Å^2^ and an SC score of 0.686 (S2 Table). JPU-G3 binds LC/A with BSA of 916 Å^2^ and a lower SC score of 0.629, which is mainly mediated by its CDR3 (**S2 Table**). The weaker binding of JPU-G3 in comparison to JPU-C1 may explain its lower protease inhibition potency. Interestingly, the CDR3 of both VHHs engage overlapping epitopes near the 160-loop of LC/A in nearly opposite orientations. As a result, the three LC/A-binding residues are in reverse orientation on JPU-C1 (residues ^101^LED^103^) and JPU-G3 (residues ^104^DEL^106^) (Fig 3A–3F).

Further structural analysis indicates that the CDR3 of JPU-C1 and JPU-G3 use a similar mechanism to interact with the SNAP25-binding residues on LC/A and therefore to preclude SNAP25 binding even though their CDR3 reaches into the anchoring site II from opposite orientations (Fig 3C and 3F). For example, L101 of JPU-C1 or L106 of JPU-G3 should prevent I192 of SNAP25 from binding to F168 of LC/A. E102 of JPU-C1 or E105 of JPU-G3 form H-bonds with T176 of LC/A and will interfere with its binding to D193 of SNAP25. Both F168 and T176 of LC/A are known to be crucial for the catalysis of SNAP25 cleavage [31]. Additionally, D103 of JPU-C1 forms salt bridges with R231 and R177 of LC/A while D104 of JPU-G3 forms a salt bridge with R231 of LC/A, both thereby interfering with LC/A binding to E194 of SNAP25. Taken together, our structural findings suggest that these two VHHs inhibit LC/A activity by blocking its binding to SNAP25 anchoring site II thereby inhibiting its subsequent cleavage.

#### VHHs targeting the anchoring site I

**Fig 4:**
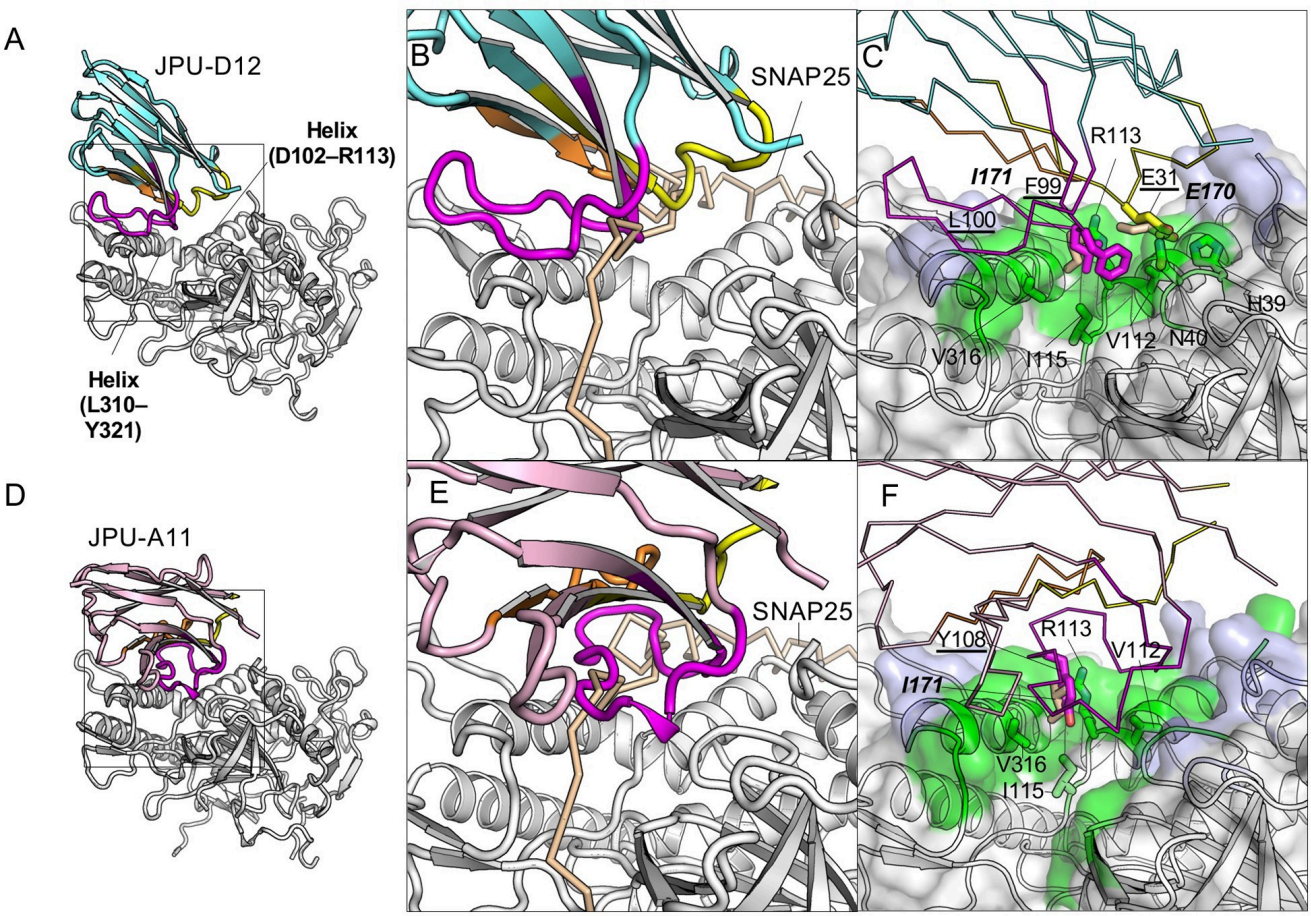
JPU-D12 and JPU-A11 block the interaction of SNAP25 to the anchoring site I of LC/A. (A, D) Cartoon representation of JPU-D12 (A) or JPU-A11 (D) in complex with LC/A. The framework region of JPU-D12 and JPU-A11 are colored cyan and pink respectively. The CDR1, 2, and 3 are colored yellow, orange, and magenta, respectively. (B, E,) Superimposition of LC/A–JPU-C1 (B) or LC/A–JPU-G3 (E) with the SNAP25(146–204)-bound LC/A (PDB ID: 1XTG). Only the VHH-bound LC/A is represented. LC/A and SNAP25 are colored as that shown in Fig 3. (C, F) The VHH residues competing with SNAP25 residues for LC/A binding are drawn as sticks. The epitopes are colored as that shown in Fig 2.

JPU-A11 and JPU-D12 bind overlapping epitopes at the anchoring site I of LC/A (Fig 4A–4F), and both are potent LC/A inhibitors (Table 1 and S3 Fig). Binding of JPU-D12 buries an interface area of 768 Å^2^ per molecule with a relatively high SC score of 0.775, while JPU-A11 interaction leads to a BSA of 984 Å^2^ and an SC score of 0.671 (S2 Table). JPU-D12–LC/A binding is strengthened by extensive 17 H-bonds and 5 salt bridges. Structural analysis showed that the CDR1 and CDR3 of JPU-D12 form a convex surface that docks to a concave pocket of LC/A primarily composed of two helices (residues L310–Y321, D102–R113) and a few neighboring loops (Fig 4A and 4B). Specifically, F99 and L100 on the CDR3 loop of JPU-D12 occupy a pocket on LC/A containing V316, I115, R113, V112 and should thereby prevent the binding of I171 of SNAP25; E31 on the CDR1 loop of JPU-D12 forms a salt bridge and a hydrogen bond with H39 and N40 of LC/A, respectively, thus making it inaccessible to E170 of SNAP25 (Fig 4C). JPU-A11 binds LC/A mainly through its CDR3 loop involving fewer electrostatic interactions (10 H-bonds and 1 salt bridge) in comparison to JPU-D12 (S2 Table). When binding to LC/A, Y108 of JPU-A11 occupies a surface hydrophobic patch on LC/A which should inhibit the binding of I171 of SNAP25 to this area (Fig 4F). The higher SC score and more extensive electrostatic interactions of JPU-D12 likely explain its greater potency than JPU-A11 to inhibit LC/A activity (S3 Fig).

#### VHHs targeting the α-exosite of LC/A

**Fig 5:**
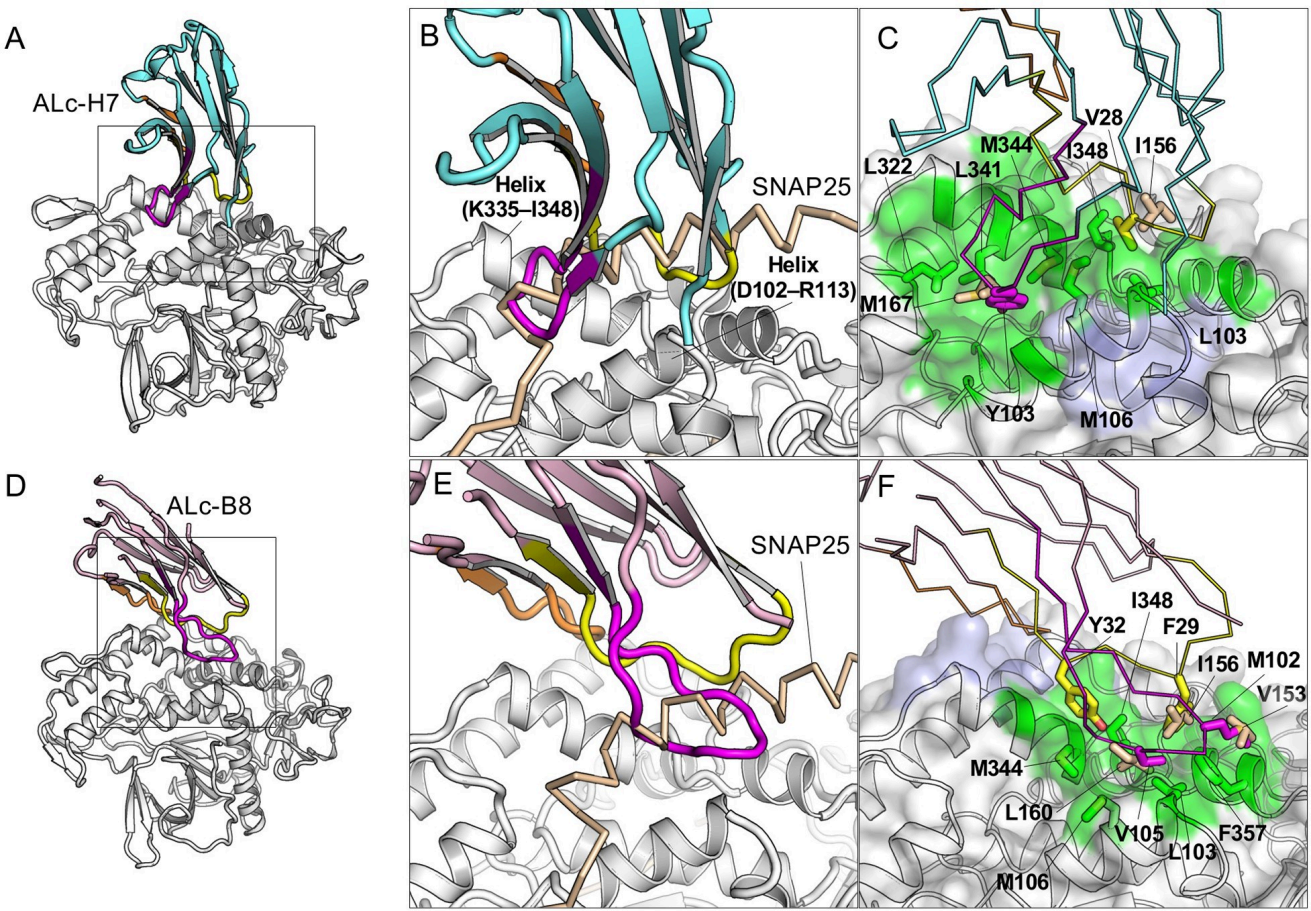
ALc-H7 and ALc-B8 block the interaction of SNAP25 to the a-exosite of LC/A. (A, D) Cartoon representation of ALc-H7 (A) or ALc-B8 (D) in complex with LC/A. The framework region of ALc-H7 and ALc-B8 are colored cyan and pink, respectively. The CDR1, 2, and 3 are colored yellow, orange, and magenta, respectively. (B, E) Superimposition of LC/A–ALc-H7 (B) or LC/A–ALc-B8 (E) with the SNAP25(146–204)-bound LC/A (PDB ID: 1XTG). Only the VHH-bound LC/A is represented. LC/A and SNAP25 are colored as that shown in Fig 3. (C, F) The VHH residues competing with SNAP25 residues for LC/A binding are drawn as sticks. The epitopes are colored as that shown in Fig 2.

Structural studies revealed that three protease-inhibitory VHHs, ALc-H7, JPU-G11 and ALc-B8, bind to the α-exosite of LC/A. ALc-H7 and JPU-G11 have identical CDR3 sequences and thus not surprisingly recognize LC/A in an almost identical manner (Fig 1A), so only ALc-H7 will be discussed further. ALc-H7 binds to LC/A and buries a molecular surface area of 715 Å^2^ and displays a high SC score of 0.764 (S2 Table). The CDR1 and CDR3 of ALc-H7 form a clamp that binds extensively to two α helices of LC/A (residues K335–I348, residues D102–R113) constituting the α-exosite (Fig 5A and 5B). V28 of the CDR1 of ALc-H7 interacts with residues L103 and I348 of LC/A which should thus prevent binding of I156 of SNAP25, while Y103 of CDR3 of ALc-H7 interacts with M106, M344, L341, L322 of LC/A and should prevent binding of M167 of SNAP25 (Fig 5C). Therefore, ALc-H7 and JPU-G11 should strongly compete with SNAP25 for binding to the α-exosite of LC/A.

The ALc-B8-binding epitope on LC/A is partially overlapping with that of ALc-H7, but ALc-B8 employs a different binding mode. The interaction of ALc-B8 with LC/A results in a BSA of 777 Å^2^ per molecule and a SC score of 0.739 (S2 Table). All CDRs of ALc-B8 contribute to LC/A recognition (Fig 5D and 5E). We found that F29, Y32, M102 and V105 of ALc-B8 bind a hydrophobic patch on LC/A that is formed by M106, L103, F357, M344 and I348 and should compete for LC/A binding to SNAP25 residues V153, I156, and L160 (Fig 5F). We noticed that a previously reported VHH Aa1 (PDB-ID: 3K3Q) has an epitope overlapping with ALc-B8 and ALc-H7 [11], but Aa1 has a smaller BSA. Together they reveal the diverse binding mechanisms that VHHs can employ for binding to the α-exosite epitope. Recent studies showed ALc-B8, when fused to an atoxic BoNT delivery vehicle, could protect mice, guinea pigs, and monkeys against BoNT/A1 intoxication even when delivered following the onset of symptoms of botulism [9,10]. These results indicate that α-exosite binding VHHs can effectively inhibit cytosolic LC/A1 activity in vivo.

### Structures and inhibition mechanisms of anti-LC/B VHHs

**Fig 6:**
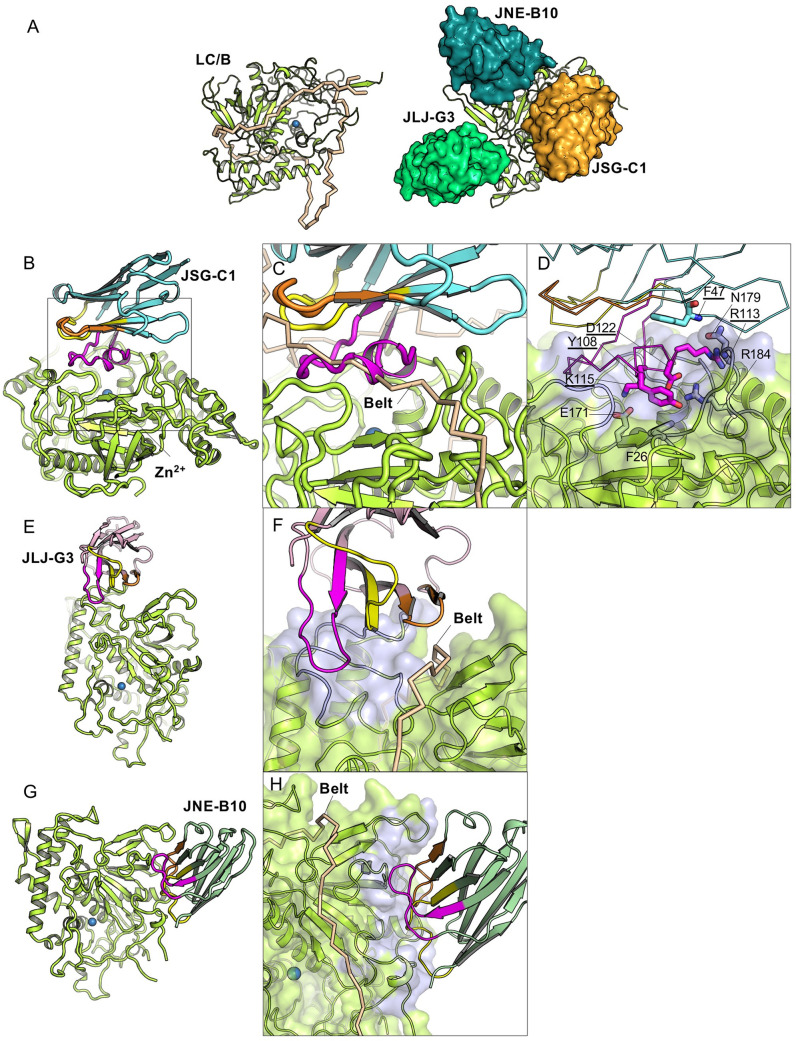
The structural basis for JSG-C1 and JLJ-G3, but not JNE-B10, to inhibit the cleavage of VAMP2 by LC/B. (A) (left) The structure of LC/B (limon cartoon) and the heavy chain belt (wheat ribbon) in the context of BoNT/B holotoxin (PDB code: 1EPW). (right) Superimposition of the LC/B-bound JSG-C1 (orange), JLJ-G3 (light green), and JNE-B10 (dark green) on the fLC/B model. (B–H) JNE-B10, JLJ-G3, and JSG-C1 occupy three distinct binding epitopes on LC/B. Cartoon representation of JSG-C1 (B), JLJ-G3 (E), and JNE-B10 (G) in complex with LC/B. The framework region of JSG-C1, JLJ-G3, and JNE-B10 are colored cyan, pink, and light green, respectively. The CDR1, 2, and 3 are colored yellow, orange, and magenta, respectively. Superposition of LC/B–JSG-C1 (C),–JLJ-G3 (F) and–JNE-B10 (H) with BoNT/B. Only VHH-bound LC/B and the heavy chain belt (wheat) of BoNT/B are drawn. The VHH-binding epitopes on LC/B are colored blue. Potential JSG-C1 residues competing with VAMP2 residues for LC/B binding are represented as sticks in panel (D).

BoNT/B is the second most prevalent cause of human botulism behind BoNT/A, and its toxicity results from cytosolic LC/B cleavage of VAMP1 or VAMP2 at ^76^QF^77^ [32]. Although the structure of LC/B–VAMP remains unresolved, the structure of the LC/F–VAMP complex suggests that VAMP likely recognizes multiple anchoring points on LC/B along the shallow grove covered by the heavy chain belt in the holotoxin (Fig 6A) [33]. Prior mutagenesis studies support the concept that, like LC/A, the substrate-binding pocket of LC/B likely aligns closely to how the heavy chain belt is associated in the holotoxin [32,34,35]. Truncation studies demonstrated that only 25 residues of VAMP2 (aa 61–85) are required for binding to LC/B while 57 residues (aa 146–202) of SNAP25 are required for LC/A binding [32,34,36].

Perhaps because of the relatively small LC/B-binding region in VAMP, competition studies with our full panel of LC/B-binding VHHs found that all protease inhibitory VHHs recognized only two non-overlapping epitopes. All of the most potent VHH inhibitors were members of the same competition group, in which JSG-C1 represents one of the most potent inhibitors of LC/B identified in this work (Table 2 and S4 Fig). For structural studies, we selected JSG-C1, as well as the previously identified JLJ-G3 [8] that is a moderate LC/B protease inhibitor binding to a distinct, non-overlapping epitope. We also included a third VHH, JNE-B10, in this study which binds at a third non-competing site and displays no protease inhibition activity, but unexpectedly provided protection against BoNT/B when delivered by an atoxic BoNT delivery vehicle to post-symptomatic animals [9] (Table 2).

The binding of JSG-C1 to LC/B buries an interface area of 1063 Å^2^ with a high SC score of 0.721 (S2 Table). Structural analysis showed that JSG-C1 binding does not cause any structural changes on LC/B (PDB code 2ETF). The binding is mainly mediated by the long CDR3 that folds into a loop with a helical structure at the tip (Fig 6B). JSG-C1 binds to a pocket close to the active site of LC/B through extensively electrostatic interactions involving 21 H-bonds and 3 salt bridges (S1 Table). More specifically, D112 and K115 of JSG-C1 form salt bridges with R184 and E171 of LC/B, respectively; Y108 of JSG-C1 interacts hydrophobically with F26 of LC/B, and R113 and F47 are hydrogen bonded with N179 of LC/B (Fig 6D). Prior studies found that all these LC/B residues involved in VHH binding are important for the catalysis of VAMP2 cleavage by LC/B [35]. Our findings thus suggest that the binding of JSG-C1 to LC/B occludes VAMP from engaging at the cleavage pocket of LC/B (Fig 6C).

JLJ-G3 is the only BoNT protease inhibitor we have identified to date that is able to bind both the isolated protease domain and the holotoxin. The crystal structure shows that JLJ-G3 binding buries an interface area of 647 Å^2^ with an SC score of 0.695 on LC/B (S2 Table and Fig 6E and 6F). As predicted by its binding to holotoxin, JLJ-G3 binds outside the heavy chain belt-binding areas which is also believed to mimic the VAMP2-binding regions. However, the CDR3 and FR2 of JLJ-G3 form a concave surface that captures two surface loops of LC/B adjacent to the VAMP2-binding groove which should cause side-to-side clashes with the N-terminal region of VAMP2 when it binds to the substrate-binding pocket of LC/B. Therefore, JLJ-G3 is able to block VAMP2 binding to LC/B through side-to-side clashes without directly occupying the VAMP2-binding interface. In contrast to JLJ-G3, the JNE-B10-binding epitope on LC/B is not involved in substrate-binding or the interaction between LC/B and the heavy chain belt (Fig 6G and 6H), which is consistent with its lack of protease inhibition activity in our assays.

### Identification of VHHs with broad BoNT/A and BoNT/B subtype specificities

**Fig 7:**
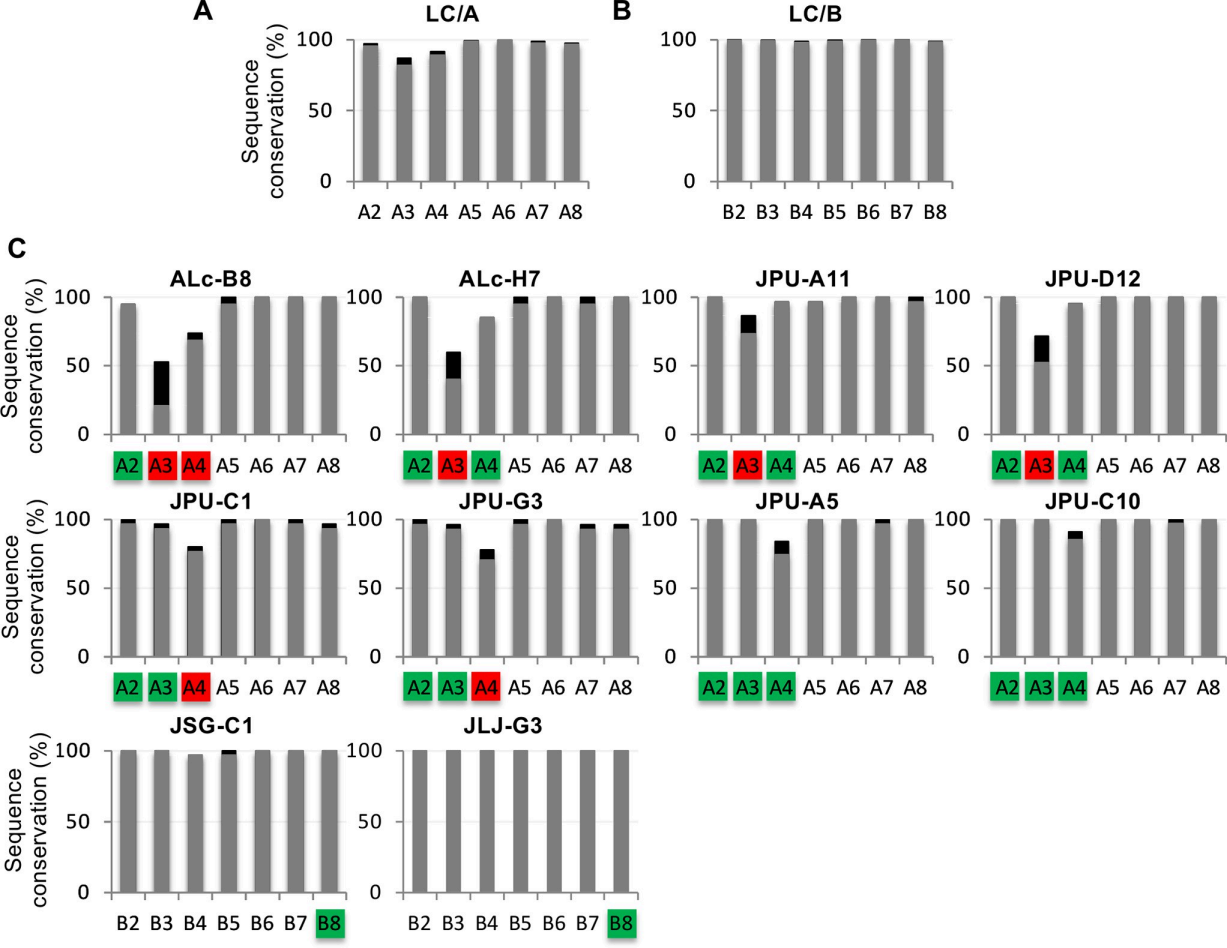
Sequence conservation of VHH-binding epitopes on LC across BoNT/A or BoNT/B subtypes. (A, B) The overall amino acid sequence identity (grey) and similarity (black) among BoNT/A or BoNT/B subtypes using A1 and B1 as the benchmarks. (C) The conservation of epitope sequences for selected LC/A- and LC/B-binding VHHs are shown. Green labels indicate comparable VHH binding to that specific subtype in comparison to A1 or B1, while red indicating weakened binding.

There are eight BoNT/A subtypes reported to date and the subtype A3 and A4 are the most diverged from A1, sharing primary sequence identity of 82.7% and 89.3%, respectively (Fig 7A). We analyze the sequence conservation of epitopes among BoNT/A subtypes for those potent inhibitor VHHs and evaluate the impact on antibody binding. The sequences near the α-exosite of A3 are particularly divergent from those in A1. It is thus not surprising that the epitopes of ALc-B8 and ALc-H7 on LC/A1 have only 21% and 40% identity with the equivalent area on LC/A3 (Figs 7C and S5A and S5B), indicating these two VHHs would bind poorly to LC/A3. These predictions were validated by dilution ELISAs which found that ALc-B8 and ALc-H7 bind to LC/A3 with EC_50_ values >125 nM (S6 Fig). In other examples, the sequences of the anchoring site I of LC/A3 have diverged significantly from that of LC/A1 (Fig 7C), suggesting JPU-A11 and JPU-D12 would bind LC/A3 poorly (S5C and S5D Fig). Furthermore, the sequences of the anchoring site II of LC/A4 are divergent from subtype A1, and our ELISA data support the structural predictions that both JPU-C1 and JPU-G3 would bind poorly to LC/A4 (S5E and S5F Fig).

In contrast to the SNAP25-binding sites discussed above, the β-exosite region on LC/A is highly conserved across different A subtypes. Therefore, the epitopes of our two β-exosite-targeting VHHs, JPU-A5 and JPU-C10, are >80% conserved in all BoNT/A subtypes (S5G and S5H Fig). Consistent with these structural findings, ELISA studies support their broad specificity for LC/A subtypes (S6 Fig). These findings indicated that these two β-exosite-binding VHHs will be excellent candidates for use in biomolecular antidotes [9,10] as a result of their high potency to inhibit the LC/A protease and their broad specificity for all known BoNT/A subtypes.

LC/B is far less variable than LC/A in nature despite the eight natural subtypes identified to date as these subtypes contain few amino acid differences as shown in Fig 7B. The subtype that is most divergent from LC/B1 is LC/B8, which has 98.2% identity. Structural studies predict that the JSG-C1 binding site on LC/B is highly conserved (only 1 conservative amino acid change in B4 and B5) and the JLJ-G3 binding site is identical across all BoNT/B subtypes (Figs 7C and S5I and S5J). Binding studies confirm that both VHHs bind effectively to both B1 and B8 subtypes (S6 Fig). Taken together, these results with LC/A and LC/B VHHs demonstrate that structural studies of VHHs in complex with their targets can have significant value in predicting the range of VHH binding to natural target subtypes.

## Discussion

Botulinum neurotoxins are dangerous biothreat agents for which no post-intoxication antidote is available, and this results in a serious public health vulnerability. In a recent development, VHHs targeting LC/A or LC/B fused to an atoxic BoNT delivery vehicle were found capable of preventing death in mice, hamsters and primates that were treated post-symptomatically after receiving a lethal dose of BoNT/A or BoNT/B [9,10]. This opens a new avenue in the development of effective biomolecular antidotes for botulism in which sdAbs such as VHHs are delivered to the cytosol of intoxicated neurons where they inhibit the BoNT protease or target its degradation. In this study, we report the identification and comprehensive structural characterization of a large panel of VHHs as antidote candidates that bind to diverse epitopes on their LC/A or LC/B targets. Furthermore, studies with these VHHs revealed diverse mechanisms by which antibodies can inhibit BoNT protease activity. The findings should contribute significantly to the further development and refinement of biomolecular antidotes, as well as conventional drugs, for treating post-symptomatic botulism paralysis.

Because VHHs typically bind to conformational epitopes [13], it is critical to maintain the structure of the target when performing VHH discovery. This is proving to be particularly important in the case of BoNT protease domains as was exemplified recently for LC/E [7]. Based on VHH recognition, the conformation of LC/E protease appeared to change substantially when the protein was coated onto plastic plates such that some VHHs recognized only coated LC/E while other VHHs recognized only antibody-captured LC/E. This ‘plasticity’ of BoNT proteases may be related to their need to partially unfold during the translocation process from the endosome into the cytosol. To minimize conformational effects during the discovery of VHHs binding to LC/A and LC/B, in this study we performed the panning and screening process using antibody-captured proteases. In both cases, the diversity of the VHH panels that were identified was substantially improved as compared to earlier efforts employing plastic-coated proteases [12]. Furthermore, most of the new VHHs displayed higher binding affinities for captured proteases compared to plastic-coated proteases, and for some VHHs this difference was dramatic (Tables 1 and 2). We thus conclude that LC/A and LC/B, like LC/E, undergo significant conformational deformity when coated to a hydrophobic plastic surface. The results highlight the importance of maintaining conformational integrity of the target when performing antibody discovery.

The availability of large panels of new VHHs binding to LC/A and LC/B identified in this study provide reagents able to probe a large portion of the surface of each protease for their functional role in SNARE protein recognition and cleavage (Figs 1 and 6). BoNT proteases are remarkably specific for their different SNARE protein substrates and this specificity is considered to be a result of the large region of interactions between these proteases and their substrates [15,33]. For LC/A, protease inhibiting VHHs were identified that bind to all four of the previously reported SNAP25-binding sites, called the α- and β-exosites and the anchoring sites I and II. Of note, JPU-A5 and JPU-C10 bind to the β-exosite of LC/A, which is a deep cleft at the active site of the protease and difficult to access by conventional Abs [37,38]. Our findings show that LC/A protease function is inhibited when a VHH can interfere with SNAP25 binding to any one of these four previously identified sites of interaction. Thus, this study strongly supports the essential roles of all four of the SNAP25-binding sites for LC/A cleavage to occur. In contrast to LC/A, we identified only two non-overlapping sites on LC/B at which protease inhibiting VHHs could bind and interfere with VAMP binding. This finding is consistent with reports that VAMP binds to LC/B through a smaller interface that is yet to be fully characterized [32,34].

Another major finding of this study is the new insight into the structure and function of the enigmatic carboxyl terminal region of LC/A. The full-length LC/A (fLC/A) including this region was previously considered as uncrystallizable because it is flexible and prone to aggregation [24]. We identified two VHHs (JPU-C10 and JPU-B9), both with epitopes that include the C-terminal region of fLC/A, which help us to determine the structures of fLC/A for the first time. We found that the LC/A C-terminus dynamically alters between at least two different conformations, which likely plays an important role in facilitating SNAP25 binding and release [21,39].

BoNTs are found in nature as at least seven different serotypes, and within each serotype are also found a substantial number of subtype variants. For therapeutic botulism antitoxins or antidotes to be broadly effective, they must be capable of protecting individuals from this diverse array of potential threats. Studies have shown that mAbs can be identified having broad specificity for natural subtype variability [5,40] and the same should be true of VHH antibodies. We previously hypothesized that structural studies of VHHs bound to BoNT targets can be used to predict their subtype specificity [41] and this study provides strong experimental support for this concept. By knowing the critical amino acids involved in the binding of many VHHs to their targets, we were able to predict their subtype specificity and then successfully verify those predictions through binding studies. We further demonstrated that, by creating large panels of VHH binding to BoNT proteases, it was possible to identify a subset of VHHs that are both highly potent and broadly active as protease inhibitors. These VHHs (e.g. JPU-A5 and JPU-C10 for LC/A, JSG-C1 for LC/B) must be considered excellent candidates as components for next generation biomolecular antidotes that should provide improved efficacy and broader variant specificity for post-exposure treatments for intoxication by the two most common threat agents, BoNT/A or BoNT/B. We note that one VHH agent, caplacizumab, has already been approved for commercialization and other VHH-based therapeutics are at late stages of development.

## Materials and methods

### Ethics statement

All procedures using alpacas were conducted in accordance with the guidelines approved by the Institute Animal Care and Use Committee at Tufts Cummings School of Veterinary Medicine under protocols G2013-85, G2017-18 and G2019-142.

### VHH discovery

Two alpacas were immunized with purified, full-length LC/A protein and two alpacas immunized with full-length LC/B as previously reported [8,12]. VHH-display phage libraries were prepared from immune PBLs obtained from each pair of immunized alpacas using standard lab procedures [42]. VHHs with affinity for native LC/A were selected by panning on LC/A immobilized by capture with the previously isolated ALc-B8 VHH [12] coated onto plastic, and this resulted in selection of the JPU series of VHHs (**Table 1**). LC/B-binding VHHs were selected in two processes. The initial VHHs were obtained by capturing LC/B to plastic with BLc-B10 VHH [12] and this led to selection of several new LC/B-binding VHHs (JND series in **Table 2**). BLc-B10 proved to poorly capture LC/B so the discovery process was repeated using JND-E4 as the capture VHH, which led to selection of many additional LC/B-binding VHHs (JSG series in **Table 2**). Screening for LC-binding VHHs was performed by ELISAs employing VHH-captured LC/A or LC/B. All VHH panning and screening was performed using standard lab procedures [42].

### Characterization of VHH binding properties

VHHs identified in the discovery process were subcloned into the pET-32 expression vector, expressed as fusions to *E*. *coli* thioredoxin and purified by standard lab procedures [42]. Binding properties were assessed by dilution ELISAs employing LC/A or LC/B proteases (0.5 or 1 μg/ml) that were coated onto Nunc MaxiSorp or Costar tissue culture plastic, or captured to Nunc MaxiSorp plastic using various protease-binding VHHs (coated at 2.5 or 5 μg/ml). LC/A subtypes 1, 2, 3 and 4, or LC/B subtypes 1 and 8, were produced as strep-tag fusion proteins (as previously described in [7]) and employed in ELISAs in which the tagged proteases were captured to streptactin plates (IBA Lifesciences). Dilution ELISAs were performed as previously described [8] and apparent binding affinities under each of the ELISA conditions were assessed by estimating the EC_50_ values, i.e. the VHH concentration that resulted in 50% peak binding. Competition assays were done as first described in [6] with some modifications provided in [7].

### Protease inhibition assays

All VHHs were tested for their potencies to inhibit LC/A and LC/B protease activity. Assays employed multiple substrates. For SNAP25 cleavage, we employed either recombinant YFP/SNAP25/CFP (BoTest A/E reporter, BioSentinel) or Repcon5 [7]. For VAMP cleavage, we employed a recombinant YFP/VAMP/CFP protein produced from an expression plasmid kindly provided by Dr. George Oyler or Repcon5. Substrate cleavage under various conditions and times was assessed by performing western blots using anti-GFP (Santa Cruz) or anti-hexahistidine (Santa Cruz) for detection of the reporter and cleavage products as previously described [7]. All assays of protease-inhibitory VHHs were repeated at least three times with different VHH: protease ratios and different times of incubation to generate the potency assessments reported in **Tables 1** and **2**.

### Cloning, expression, and purification of recombinant proteins for crystallization

LC/B(M1–S425), LC/B(M1–K441), anti-BoNT/A VHHs (JPU-G11, JPU-D12, JPU-A11, JPU-C1, JPU-G3, JPU-A5, JPU-B9, and JPU-C10), and anti-BoNT/B VHHs (JNE-B10, JLJ-G3, and JSG-C1) were cloned into pGEX-6p-1 for expression following the N-terminal GST and a PreScission cleavage site. LC/A(M1–T420), LC/A (P2–K438), ALc-H7, ALc-B8, ciA-D12, and ciA-F12 were expressed and purified as described previously [8,12,43].

GST-tagged LC/A, LC/B, and VHHs (JPU-G11, JPU-D12, JPU-A11, JPU-C1, JPU-G3, JPU-B9, JPU-C10, JNE-B10, JLJ-G3, and JSG-C1) were expressed in *E*. *coli* strain BL21-Star (DE3) (Invitrogen). GST-tagged JPU-A5 was expressed in the *E*. *coli* strain Origami B (DE3) (Novagen). Bacteria were cultured at 37°C in LB medium containing ampicillin. Temperature was reduced to 18°C when OD_600_ reached ~0.6. Expression was induced with 1 mM IPTG and continued at 18°C for 16 hr. Cells were harvested by centrifugation and stored at –20°C until use.

For protein purification, bacteria were re-suspended in a buffer containing 50 mM Tris (pH 8.0), 400 mM NaCl, and 0.4 mM PMSF and lysed by sonication. All GST-tagged proteins were purified using Glutathione Sepharose 4B resins (GE Healthcare) in 50 mM Tris (pH 8.0), 400 mM NaCl, and eluted from the resins after on-column cleavage using PreScission protease. The proteins were further purified by Superdex-200 Increase or Superdex-75 SEC in 10 mM HEPES (pH 7.4) and 150 mM NaCl. The LC–VHH complexes were made by mixing the purified LC and VHHs at a molar ratio of 1:1.5 for 1 hr on ice, followed by purification using Superdex-200 Increase SEC (10 mM HEPES, pH 7.4, 150 mM NaCl, and 5 μM ZnSO_4_). Each protein complex was concentrated to ~5 mg/ml using Amicon Ultra centrifugal filters (Millipore) and stored at –80°C until further characterization or crystallization.

### Crystallization

Initial crystallization screens were performed using a Gryphon crystallization robot (Art Robbins Instruments) and high-throughput crystallization screen kits (Hampton Research and Qiagen). Extensive manual optimizations were performed at 18°C when proteins were mixed with reservoir solution at 1:1 ratio.

1. The best single crystals of sLC/A–JPU-A5–ALc-H7–JPU-C1–JPU-D12–ciA-F12 were grown by the sitting-drop vapor diffusion method at a protein concentration of 8 mg/ml with a reservoir solution containing 20% 2-propanol, 0.1 M sodium citrate, 20% PEG4000, pH 5.6.
2. Crystals of fLC/A–ALc-B8–JPU-C10–JPU-G3–JPU-D12–ciA-F12–ciA-D12 were obtained by the sitting-drop vapor diffusion method at a protein concentration of 6 mg/ml with a reservoir solution containing 2.4 M sodium malonate, 0.1 M Bis-Tris, pH 7.0.
3. Crystals of fLC/A–JPU-B9–JPU-A11–JPU-G11 were grown by the sitting-drop vapor diffusion method at a protein concentration of 5 mg/ml with a reservoir solution containing 25% PEG 4000, 0.1 M sodium cacodylate, pH 6.5.
4. Crystals of sLC/B–JLJ-G3–JNE-B10 were obtained by the sitting-drop vapor diffusion method at a protein concentration of 5 mg/ml with a reservoir solution containing 0.2 M KI, 25% PEG 4000, 0.1 M MES, pH 6.5.
5. Crystals of fLC/B–JSG-C1 were obtained by the sitting-drop vapor diffusion method at a protein concentration of 5 mg/ml with a reservoir solution containing 20% PEG 4000, 0.15 M ammonium sulfate, 0.1 M Hepes, pH 7.0.

### Data collection and structure determination

All crystals were cryoprotected in their original mother liquor supplemented with 20–25% (v/v) ethylene glycol. The X-ray diffraction data for the crystals were collected at 100 K at the NE-CAT beamline 24-ID-E, Advanced Photon Source (APS). The data were processed with iMOSFLM [44] or XDS as implemented in RAPD (https://github.com/RAPD/RAPD) [45]. Data collection statistics are summarized in **S2 Table**. Structures of the sLC/A–JPU-A5–ALc-H7–JPU-C1–JPU-D12–ciA-F12, fLC/A–ALc-B8–JPU-C10–JPU-G3–JPU-D12–ciA-F12–ciA-D12, and fLC/A–JPU-B9–JPU-A11–JPU-G11 complexes were determined by molecular replacement using the Phaser software [46] with LC/A (PDB code 1XTF) [15] and the homology models of VHHs that were built based on a VHH in PDB 5L21 [47] as the search models. Structures of the complexes were solved by molecular replacement with Phaser using LC/B (PDB code 2ETF) and the homology models of the VHHs as the search models. Manual model building and refinement were performed in COOT [48], PHENIX [49], and CCP4 suite [50] in an iterative manner. The refinement progress was monitored with the free R value using a 5% randomly selected test set [51]. The structures were validated through MolProbity [52] and showed excellent stereochemistry. Structural refinement statistics are listed in **S2 Table**. All structure figures were prepared with PyMol (http://www.pymol.org).

## Supporting information

S1 Table Data collection and refinement statistics. (PDF)

S2 Table Summary of LC-VHH binding interface information. (PDF)

S1 Fig Sequence alignment of LC/A-binding VHHs. Amino acid sequences of all of the LC/A-binding VHHs studied in this report. Sequences are aligned to conserved framework regions and CDRs are indicated.
(PDF)

S2 Fig Sequence alignment of LC/B-binding VHHs. Amino acid sequences of all of the LC/B binding VHHs studied in this report. Sequences are aligned to conserved framework regions and CDRs are indicated.
(PDF)

S3 Fig Western blot assessment of VHH potency to inhibit LC/A protease activity. LC/A protease was incubated with the BoTest A/E reporter in the presence or absence of VHHs at a molar ratio of 5:1 VHH:LC/A. Incubations were performed at 37°C for either 10 or 60 minutes as indicated, and the reaction was terminated by boiling in SDS sample buffer. An equal aliquot of each sample was analyzed by performing western blots and the substrate and products were detected by HRP/anti-GFP antibodies.
(PDF)

S4 Fig Western blot assessment of VHH potency to inhibit LC/B protease activity. LC/B protease was incubated with a recombinant YFP/VAMP/GFP reporter in the presence or absence of VHHs at a molar ratio of 2:1 VHH:LC/B. Incubations were performed at 37°C for either 60 or 180 minutes as indicated, and the reaction was terminated by boiling in SDS sample buffer. An equal aliquot of each sample was analyzed by performing western blots and the substrate and products were detected by HRP/anti-GFP antibodies.
(PDF)

S5 Fig Structural predictions as to cross-specificity of VHHs to bind to the known LC/A or LC/B subtypes. Sequence conservation of VHH-binding epitopes across BoNT/A or BoNT/B subtypes. (A–H) LC/A or (I–J) LC/B is drawn in surface. Identical, conserved, semi-conserved, and variable residues at the LC–VHH interface are colored, green, blue, purple, and red, respectively. (Right) The amino acid sequence alignment among BoNT/A or BoNT/B subtypes performed using Clustal Omega. Only the VHH-binding epitopes are represented.
(PDF)

S6 Fig Dilution ELISAs to assess the cross-specificity of VHHs for selected LC/A or LC/B subtypes. Recombinant LC/A1, /A2, /A3 and /A4 and LC/B1 and /B8, each strep-tag fusion proteins, were captured to streptactin plates and VHH binding dilution ELISAs were performed as described in the Materials and Methods. The name of the VHH tested in each ELISA plot is indicated above the data. VHH binding data to each subtype is indicated in different colors and plotted as a function of VHH concentration.
(PDF)
