# Supplementary material for: Probing the structure and function of the protease domain of botulinum neurotoxins using single-domain antibodies
Source: PLoS Pathog. 2022 Jan 6;18(1):e1010169. doi: 10.1371/journal.ppat.1010169 (PMC8769338; doi:10.1371/journal.ppat.1010169)
Supplement: S1 Table — (PDF) [file ppat.1010169.s001.pdf]

Table S1. Data collection and refinement statistics.

	sLC/A–JPU-A5–ALc-H7–JPU-C1–JPU-D12–ciA-F12 PDB ID: 7L6V	fLC/A–ALc-B8–JPU-C10–JPU-G3–JPU-D12–ciA-F12–ciA-D12 PDB ID: 7M1H	fLC/A–JPU-B9–JPU-A11–JPU-G11 PBD ID: 7LZP
**Data collection**			
Space group	I 2 2 2	P 2 21 21	P 61 2 2
Cell dimensions			
a, *b*, *c* (Å)	115.90, 157.65, 173.00	77.59, 93.98, 231.75	132.60, 132.60, 413.03
α, *β*, *γ* (°)	90, 90, 90	90, 90, 90	90, 90, 120
Resolution (Å)	161.52–2.01 (2.04–2.01)[a]	115.87–2.78 (2.88–2.78)	137.68–2.86 (2.96–2.86)
R_{pim}	0.089 (1.220)	0.082 (0.834)	0.088 (0.893)
CC1/2	0.995 (0.352)	0.996 (0.527)	0.998 (0.405)
I/σ(I)	7.5 (0.9)	11.1 (1.1)	12.2 (1.1)
Completeness (%)	98.6 (98.0)	99.6 (97.2)	99.6 (96.3)
Redundancy	2.8 (2.7)	6.7 (6.4)	9.7 (10.0)
**Refinement**			
Resolution (Å)	116.52–2.01	115.87–2.78	67.06–2.86
No. reflections	102647	43498	50380
Reflections used for R_{free}	5109	2167	2533
R_{work}/R_{free}	0.197 / 0.234	0.217 / 0.249	0.235 / 0.273
No. atoms	8494	8918	12136
Protein	7965	8867	12130
Ligand/ion	9	1	4
Water	520	50	2
B-factors	48.37	77.15	81.97
Protein	48.48	77.28	81.98
Ligand/ion	51.84	46.91	53.15
Water	46.59	54.84	47.45
R.m.s. deviations			
Bond lengths (Å)	0.006	0.002	0.003
Bond angles (°)	0.80	0.462	0.584

	sLC/B–JLJ-G3–JNE-B10 PDB ID: 7T5F	fLC/B–JSG-C1 PDB ID: 7NA9
Data collection		
Space group	C 1 2 1	P 21 21 21
Cell dimensions		
a, *b*, *c* (Å)	149.32, 88.28, 114.48	61.42, 97.02, 101.90
α, *β*, *γ* (°)	90, 97.03, 90	90, 90, 90
Resolution (Å)	113.62–2.60 (2.74–2.60)	101.90–1.76 (1.79–1.76)
R_{pim}	0.069 (0.457)	0.033 (0.328)
CC1/2	0.99 (0.52)	0.999 (0.852)
I/σ(I)	6.3 (1.5)	20.1 (2.8)
Completeness (%)	96.3 (95.2)	99.5 (92.4)
Redundancy	2.8 (2.9)	6.7 (5.6)
**Refinement**		
Resolution (Å)	75.84–2.6	52.60–1.76
No. reflections	45121	61145
Reflections used for R_{free}	2280	3110
R_{work}/R_{free}	0.19 / 0.22	0.158 / 0.200
No. atoms	10467	5179
Protein	10329	4597
Ligand/ion	8	13
Water	130	569
B-factors	53.70	26.79
Protein	53.8	25.71
Ligand/ion	64.8	30.79
Water	42	35.43
R.m.s. deviations		
Bond lengths (Å)	0.01	0.016
Bond angles (°)	1.28	1.325

One crystal was used for each structure.

[a]Statistics for the highest-resolution shell are shown in parentheses.
