# Supplementary material for: Probing the structure and function of the protease domain of botulinum neurotoxins using single-domain antibodies
Source: PLoS Pathog. 2022 Jan 6;18(1):e1010169. doi: 10.1371/journal.ppat.1010169 (PMC8769338; doi:10.1371/journal.ppat.1010169)
Supplement: S2 Table — (PDF) [file ppat.1010169.s002.pdf]

Table S2. Summary of LC–VHH binding interface information

| | VHH | PBD ID | Sc | BSA ($A^2$) Total | BSA ($A^2$) CDR1 | CDR2 | CDR3 | HB* | SB# |
|---|---|---|---|---|---|---|---|---|---|
| Anti-LC/A VHH | JPU-C10 | 7M1H | 0.731 | 1214.8 | 195.4 | 169.9 | 530.9 | 17 | 2 |
| | JPU-A5 | 7L6V | 0.686 | 1033.1 | 106.5 | 72.9 | 613.1 | 11 | 7 |
| | JPU-B9 | 7LZP | 0.639 | 936.4 | 198.4 | 228.2 | 108.9 | 6 | 6 |
| | JPU-C1 | 7L6V | 0.686 | 1010 | 225.3 | 235.7 | 318 | 23 | 3 |
| | JPU-G3 | 7M1H | 0.629 | 916.3 | 0 | 25.4 | 562.1 | 16 | 4 |
| | JPU-D12 | 7L6V | 0.775 | 768.1 | 172.3 | 77.4 | 410.2 | 17 | 5 |
| | JPU-A11 | 7LZP | 0.671 | 983.6 | 43.4 | 47.9 | 775.8 | 10 | 1 |
| | JPU-B8 | 7M1H | 0.739 | 776.8 | 167.6 | 243.9 | 207.1 | 6 | 1 |
| | JPU-H7 | 7L6V | 0.778 | 714.9 | 163.5 | 23.8 | 311.3 | 9 | 0 |
| Anti-LC/B VHH | JNE-B10 | 5L2C | 0.658 | 898 | 294.3 | 272.1 | 132.3 | 7 | 3 |
| | JLJ-G3 | 5L2C | 0.695 | 647.1 | 18.6 | 35 | 172 | 7 | 2 |
| | JSG-C1 | 7NA9 | 0.721 | 1063.1 | 28.8 | 73.7 | 692 | 21 | 3 |

* HB - Number of hydrogen bonds
# SB - Number of salt bridges
