# Supplementary material for: Probing the structure and function of the protease domain of botulinum neurotoxins using single-domain antibodies
Source: PLoS Pathog. 2022 Jan 6;18(1):e1010169. doi: 10.1371/journal.ppat.1010169 (PMC8769338; doi:10.1371/journal.ppat.1010169)
Supplement: S1 Fig — Amino acid sequences of all of the LC/A-binding VHHs studied in this report. Sequences are aligned to conserved framework regions and CDRs are indicated. (PDF) [file ppat.1010169.s003.pdf]

Supplementary Figure 1

```
                                 CDR1                CDR2                                        CDR3
ALc-B8   SGGGLVQPGGSLRLSCAASGSIFSIYAMGWYRQAPGKQRELVAAISS-YGSTNYADSVKGRFTISRDNAKNTVYLQMNSLKPEDTAVYYCNA--------DIATMTAVGGFDYWGQGTQVTVSS
ALc-H7   SGGGSVQPGGSLRLSCAAIGSVFTMYTTAWYRQTPGNLRELVASITD-EHRTNYAASAEGRFTISRDNAKHTVDLQMTNLKPEDTAVYYCKL------------EHDLGYYDYWGQGTQVTVSS
ciA-D1   SGGGLVQPGGSLRLSCATSGFTLEYYAIGWFRQAPGKGREGVACMNSSGGGTNYADSVKGRFTISRDNAKKMVYLQMNSLKSEDTAVYYCVV-----DDFRCGSRWAAYLRSSWGQGTQVTVSS
ciA-D12  SGGGLVQPGGSLRLSCVVSGSDFNTYIMGWYRQVPGKPRELVADITT-EGKTNYGGSVKGRFTISRDNAKNTVYLQMFGLKPEDAGNYVCNA-----DWKMGAWTAGDYGIDYWGKGTLVTVSS
ciA-F12  SGGGLVQPGGSLRLSCAASGFTLGSRYMSWVRQAPGEGFEWVSSIEP-SGTAWDGDSAKGRFTTSRDDAKNTLYLQMSNLQPEDTGVYYCAT---------GYRTDTRIPGGSWGQGTQVTVSS
ciA-H7   SGGGLVQVGGSLRLSCVVSGSDISGIAMGWYRQAPGKRREMVADIFS-GGSTDYAGSVKGRFTISRDNAKKTSYLQMNNVKPEDTGVYYCRL---------------YGSGDYWGQGTQVTVSS
JPU-A1   SGGGLVQPGGSLRLSCAASGFTLDDYAIGWFRQVPGKEDEGVSCMSR-SGDTYYPHSVKGRFTISVDNAKNTMYLQMNNLKPEDTAVYYCAI-----DFPPVRPMCIQAAPKKRSRGTQVTVSA
JPU-A5   TGGGLVQAGGSLRLSCTASGADFSFYAMGWYRQTPGNSRELVAVMNL-NGVISYGDSARGRFDISRDGTKNIVFLQMNSLKPEDTGVYYCNG-------MRLYTRGSVRHPESWGQGIQVTVSS
JPU-A11  TGGGLVQAGDSLTLSCAATGRTLDYYALGWFRQVPGNKREFVAAINWLGGSTYYADSVRGRFTLSRDNSKSTLYLNMNNLIPDDTAVYYCAADFSIAYSGTYPPAYAEYDYDYWGQGTQVTVSS
JPU-B5   S-GGLVQPGGSLKLSCAHSGSPLSIWVMGWYRQAPGKQRELVALINL-NGITSYGDSVKGRFTISRDYAENTAYLQMNSLKFEDTAVYYCNA-------EPLGPRGKKSGKEYWGTGTQVTVSL
JPU-B9   T-GGALVQPGQSLTLSCTTSENVFGIYGMAWLRQAPGRQRELVASITSRGTAHYHDSVKGRFTISRESGKTTAYLQTTSVNPEDTAIYYCNS------------------GPYWGQGTQVTVSS
JPU-C1   SGGGLVQPGGSLRLSCAASGFTFNRYVIRWYRQAPGKERELVAGISRSGDSGRYVDSVKGRFTISRDNDKNMAYLQMSSLKPDDTAVYYCSA-------------LNLEDMEYWGQGTQVTVSS
JPU-C10  SGGGLVQPGGSLRLSCAASGNIFSIYYMGWYRQAPGKQREMVAIINS-NGITNYGDFVKGRFTISRDNAENSAYLQMNNLTPEDTAVYYCNA-------GKLRRTTGWGLDDYWGQGTQVTVSS
JPU-D12  SGGGTVQPGGTLRLSCAASGFTLDEYAIGWFRQAPGKEREGVSCISS-SASISYADSVKGRFTISRDNAKNTVYLTMNSLKPEDTGVYYCAR----AFLACGPVAGWGTEYDYWGQGTQVTVSS
JPU-G3   TGGGLVQPGGSLRLSCTASTTISDFYSMGWFRQTPGNQRELVAIVRR-GGDTKSGDSVKGRFTISRDNTRSTVYLQMDNLKPEDTAVYYCYA--------NLQKSSDELGPYYWGQGTQVTVSS
JPU-G7   SGGGLVQSGGSLRLSCAASLLTLEYYAIGWFRQAPGKEREGVSCTGSSGGSTVYIDSVKGRFTVVRDNAKNMVYLQMDNLQPEDTAVYYCAA-----DDLRCGRGWSSYFRGSWGQETQVTVSS
JPU-G11  TGGGLVQPGGSLRLACVASESVFEMYTVAWYRQAPGKQRELVAGITD-EGRTNYADFVKGRFTISRDNSKKTVHLQMDNLNPEDTAVYYCKL------------EHDLGYYDYWGQGTQVTVSS
JPU-G12  SGGGLVQPGGSLRLSCAASGLTLDYYAIGWFRQAPGKEREGVSCISSGSSMSIHADSVKGRFTISRDNAKNTVYLQMNSLKPEDTAVYYCAA---DDFTCGSRWSDWAHTFGFWGQGTQVTVSS
JPU-H7   S-GGLVQPGGSLTLSCVVSGGIFSTYIMGWYRQVPGRQREMVATISN--HTTDYADFVQGRFTISRDIAKKAVYLQMHSLKPDDTGRYVCNA-----DWMVGAWTAGDYGVDYWGKGILVTVSS
```
