# Supplementary material for: Probing the structure and function of the protease domain of botulinum neurotoxins using single-domain antibodies
Source: PLoS Pathog. 2022 Jan 6;18(1):e1010169. doi: 10.1371/journal.ppat.1010169 (PMC8769338; doi:10.1371/journal.ppat.1010169)
Supplement: S2 Fig — Amino acid sequences of all of the LC/B binding VHHs studied in this report. Sequences are aligned to conserved framework regions and CDRs are indicated. (PDF) [file ppat.1010169.s004.pdf]

Supplementary Figure 2

```
                                  CDR1                     CDR2                                                  CDR3
BLc-B10   SGGGMVQPGGSLRLSCAASGFT--FSTYDMSWVRQAPGKGPEWVSIINAGGGSTYYAASVKGRFAISRDNAKNTLYLQMNNLKPEDTALYYCAR--VASYYCRGYVCSPPEFDYWGQGTQVTVSS
JLJ-F9    SGGGLVQAGGSLRLSCAPSRLT--LDFFAIAWFRQAPGKEREGVSCISSHDGSTYYTDSVKGRFTISKDNAKNTVYLQMNSLKPEDTAVYYCAL----DHNVGTCQLTQAEYDYWGQGTQVTVSS
JLJ-G3    SGGGLVQSGGSLRLSCAASGSI--DSLYHMGWYRQAPGKERELVARVQD-GGSTAYKDSVKGRFTISRDFSRSTMYLQMNSLKPEDTAIYYCAA-----------KSTISTPLSWGQGTQVTVSS
JNE-B10   SGGGLVQPGGSLRLSCAASGFP--FHAYYMSWVRQAPGKGLEWVSHIGNGGIITRYADSVKGRFTISRDNAKNTLYLQMTNLKPEDTALYYCTL-----------GTRDDLGPERGQGTQVTVSS
JND-A12   TGGGLVQAGGSLGLSCAASGLS--FNWYDVGWFRQAPGKEREFVASRSSGGGSTYYGDSVKGRFSISTDNAKNTAYLQMNSLKPEDTAVYYCAADWTGRAGFSVGYYRPDEYDYWGQGTQVTVSE
JND-B4    TGGGLVQPGGSLRLSCVASGFT--LDSYAIGWFRQAPGKEREGVSCMSSGDGSTYYTNSVKGRFTISRDNAQNTVYLQMNSLKPEDTAVYYCAA---DGFDYCSAYVPGRGMNYSGKGTLVTVSS
JND-C7    TGGGLVQPGGSLRLSCAGSGFT--LDNYAVGWFRQAPGKEREGVSCISSSDDNTDYSDSVKGRFTISRDNAKDTVYLQMNSLKPEDTAIYYCAA--ESPTFGFSCTVATDPYDYWGQGTQVTVSS
JND-E4    TGGGLVQPGGSLRLSCAASGFT--LDGYAAGWFRQAPGKERELVSWISSTDGSTYYAASVKGRFTVSRDNAKNTVYLQMNSLKPEDTAVYYCTA-------GLGLDVSDYVYDYWGQGTQVTVSS
JND-E5    S-GGLVQPGGSLRLSCAASGFT--LDYYGIGWVRQAPGKEREEVSCITS-GGLTNYPDSVKGRFTISRDNAKNTVYLQMNSLKPEDTAVYYCAI-------DRVGVCAMEDFGSWGQGTQVTVSS
JND-E9    TGGGLVQAGDSLRLSCAASGRT--FNYYAMAWFRQAPGKEREFVAFINWSGDSTYYAGSVKGRFTISRDNAKNTVYLQMNNLKPEDTAVYSCAA-----EFGTFSYLQGDDYSYWGQGTQVTVSS
JND-F3    SGGGLVQAGGSLRLSCAASGRS--FSSYRMGWFRQAPGKERELVAGISWSGSSTWYADSVKGRFTISRDNAKNTVYLQMNSLKPEDTAVYYCAA------DGLGTDWSDAIWDYWGQGTQVTVSS
JSG-B8    SGGGLVQAGGSLRLSCAVSGRM--FNEYRMGWFRQAPGKEREFVSAINWGAQIPYYADSVKGRFTISRDSAENTVYLQMNSLKPEDTAVYYCAA----DWGYGSSPHQDKEYDYWGQGTQVTVSS
JSG-B10   SGGGLVQAGGSLRLSCAASGRT--FSDYAMGWFRQAPGKERVFVAAVDWSGSSRLYAESVEGRFTISRDNSKNTVYLQMNTLKPEDTAVYYCAA------ARNRWSSEISSYDYWGQGTQVTVSS
JSG-C1    SGGGLVQTGGSLRLSCAASGRT--FRRNTMGWFRQAPGKVREFVAAISWSGDRTYCADSVKGRFTISRDNAKNTVDLLMNSLKPEDTAIYYCAADGTASVFNSYASADRNKYNYWGQGTQVTVSS
JSG-F6    SGGGLVQAGDSLRLSCAASIRT--FSTSTTAWFRQAPGKEREFVARISGSDPVTYYTDSVRGRFTISRDNAKSTAYLQMNSLKPEDTGVYYCAT-------VRIKGGSEFSYHYWGQGTQVTVSS
JSG-G1    TGGGLVQAGGSLRLSCAASEST--FSINAIGWYRQAPGKQRELVAHIST-SGRTRYADSVKGRFTISRDNAWNTVFLQMISLKPEDTAVYYCNA------EGYSTWPEDRYLELWGQGTQVTVSP
JSG-G10   TGGGLVQAGGSLRLSCTASGRTFSSYRMGMGWFRQAPGKEREFVATVNWSGGTTYYADSVKGRFTISRDNAKNTVYLQMNSLKPEDTAVYYCAA------GRGSESYTSSRYNYWGQGTQVTVSS
JSG-G11   SGGGLVQPGGSLRLSCAASGFT--LDDYAIGWFRQAPGKEREAVSCISIRDGRTHYADSVKGRFTISRDNAKNTVYLQMNSLKPEDTAVYYCAA-----GQRSMAYVCSNRFGSWGQGTQVTVSS
```
