# Supplementary figures and images for: Probing the structure and function of the protease domain of botulinum neurotoxins using single-domain antibodies
Source: PLoS Pathog. 2022 Jan 6;18(1):e1010169. doi: 10.1371/journal.ppat.1010169 (PMC8769338; doi:10.1371/journal.ppat.1010169)

Supplementary Figure 3

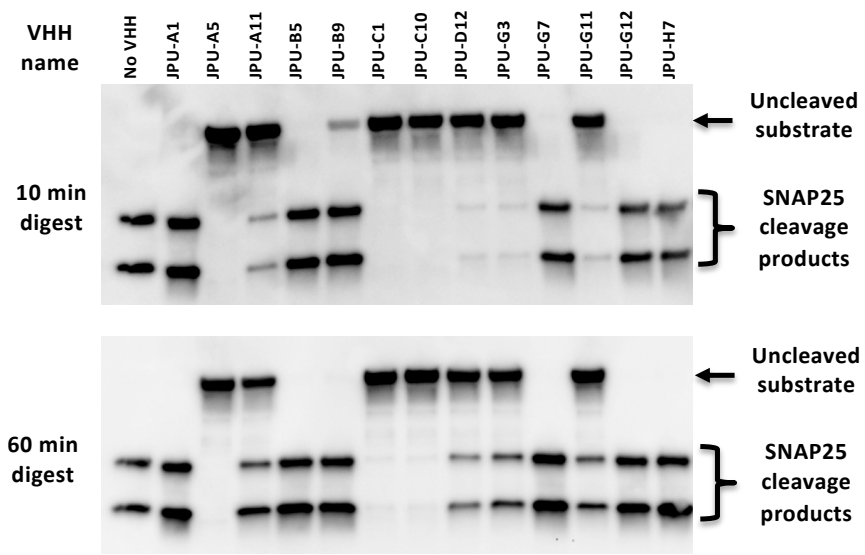

Supplement: S3 Fig — LC/A protease was incubated with the BoTest A/E reporter in the presence or absence of VHHs at a molar ratio of 5:1 VHH:LC/A. Incubations were performed at 37°C for either 10 or 60 minutes as indicated, and the reaction was terminated by boiling in SDS sample buffer. An equal aliquot of each sample was analyzed by performing western blots and the substrate and products were detected by HRP/anti-GFP antibodies. (PDF) [file ppat.1010169.s005.pdf]

Supplementary Figure 4

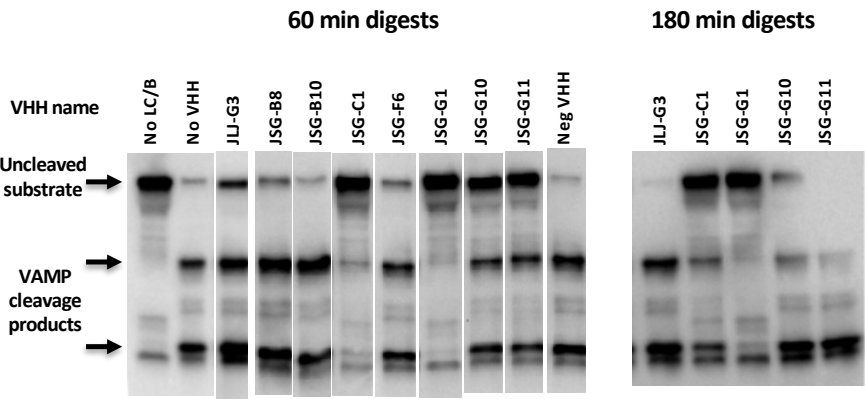

Supplement: S4 Fig — LC/B protease was incubated with a recombinant YFP/VAMP/GFP reporter in the presence or absence of VHHs at a molar ratio of 2:1 VHH:LC/B. Incubations were performed at 37°C for either 60 or 180 minutes as indicated, and the reaction was terminated by boiling in SDS sample buffer. An equal aliquot of each sample was analyzed by performing western blots and the substrate and products were detected by HRP/anti-GFP antibodies. (PDF) [file ppat.1010169.s006.pdf]

Supplementary Figure 6

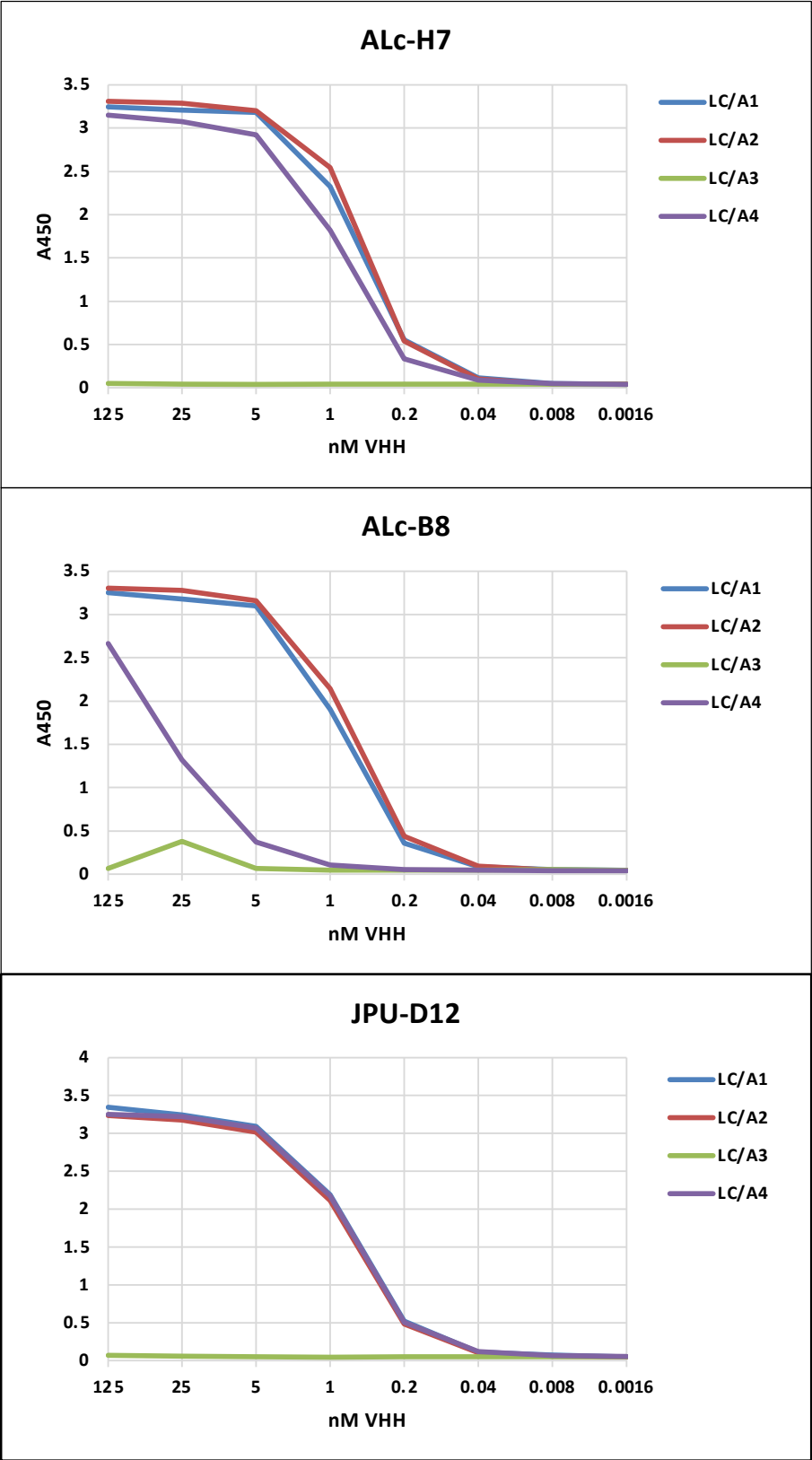

### JPU-A11

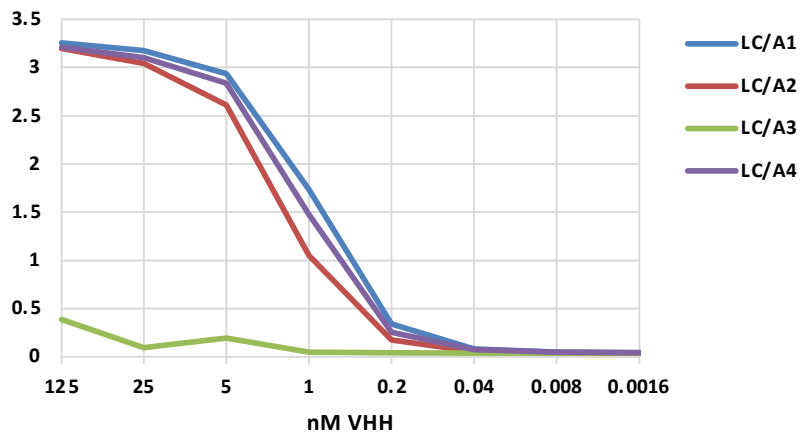

### JPU-G3

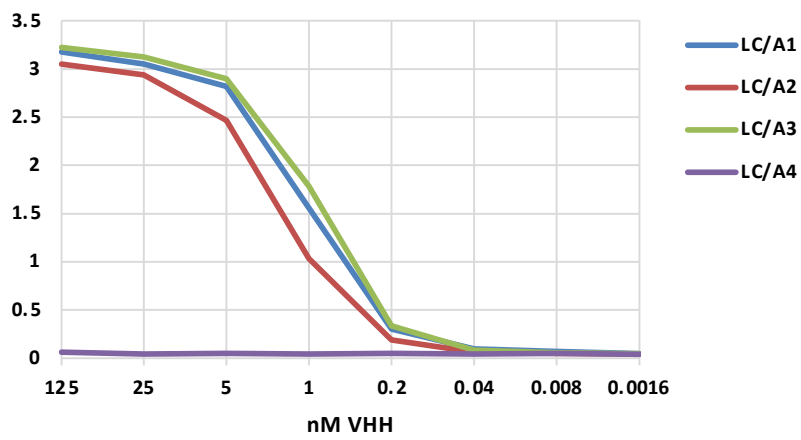

### JPU-C1

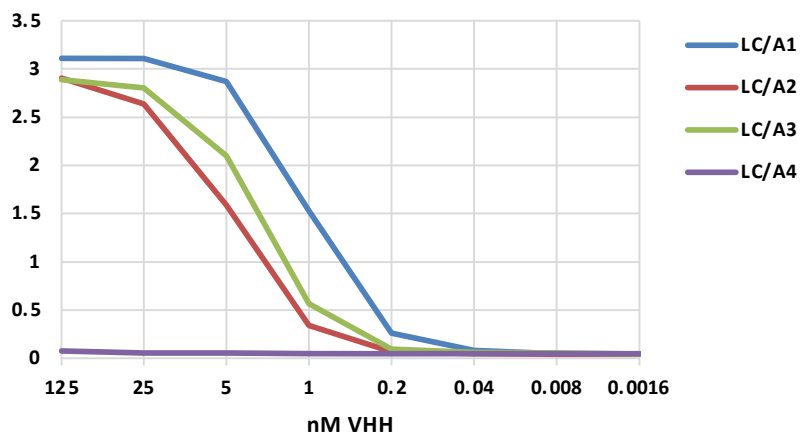

### JPU-C10

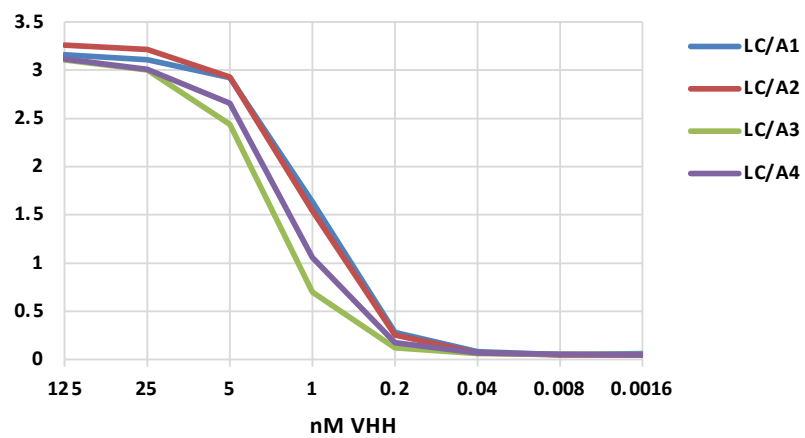

### JPU-A5

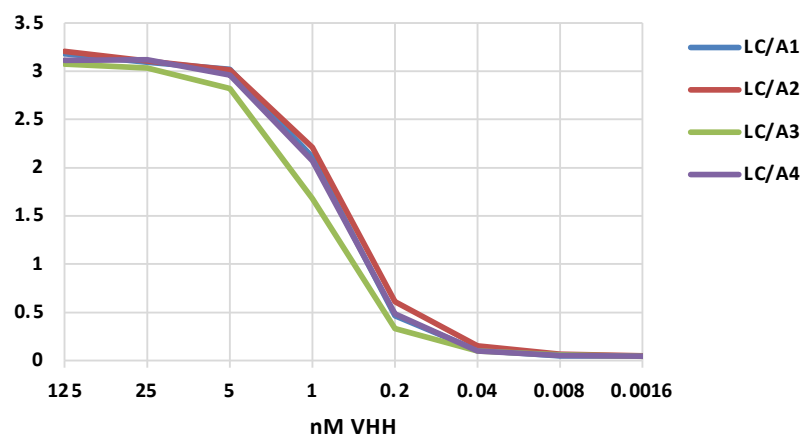

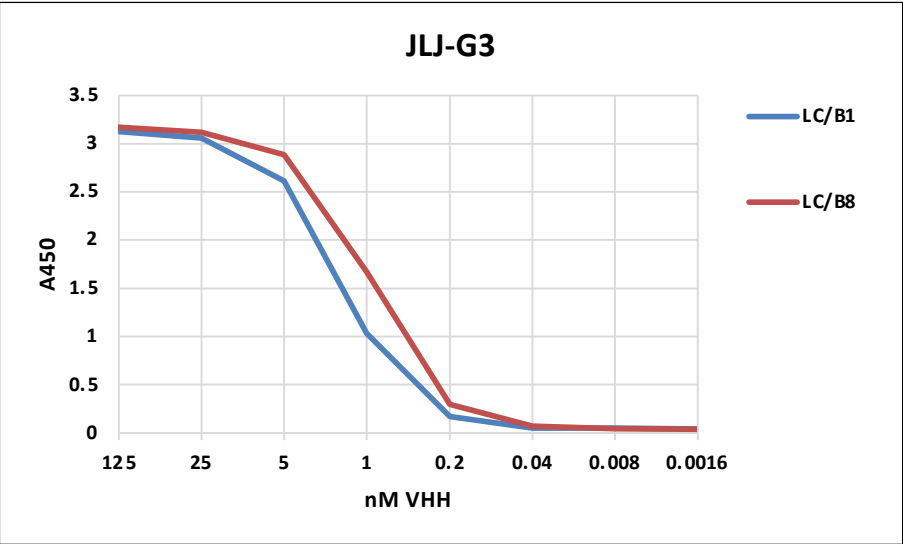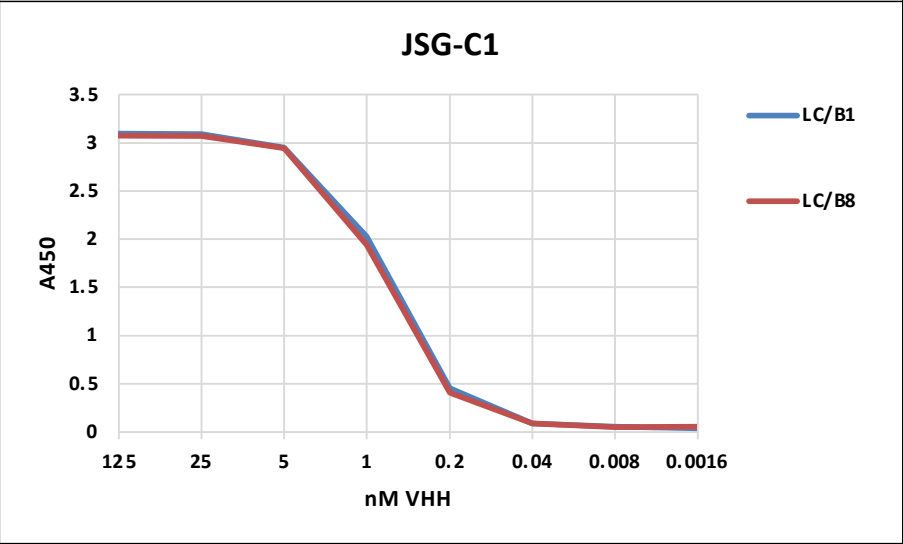

Supplement: S6 Fig — Recombinant LC/A1, /A2, /A3 and /A4 and LC/B1 and /B8, each strep-tag fusion proteins, were captured to streptactin plates and VHH binding dilution ELISAs were performed as described in the Materials and Methods. The name of the VHH tested in each ELISA plot is indicated above the data. VHH binding data to each subtype is indicated in different colors and plotted as a function of VHH concentration. (PDF) [file ppat.1010169.s008.pdf]
