# Supplementary material for: Probing the structure and function of the protease domain of botulinum neurotoxins using single-domain antibodies
Source: PLoS Pathog. 2022 Jan 6;18(1):e1010169. doi: 10.1371/journal.ppat.1010169 (PMC8769338; doi:10.1371/journal.ppat.1010169)
Supplement: S5 Fig — Sequence conservation of VHH-binding epitopes across BoNT/A or BoNT/B subtypes. (A–H) LC/A or (I–J) LC/B is drawn in surface. Identical, conserved, semi-conserved, and variable residues at the LC–VHH interface are colored, green, blue, purple, and red, respectively. (Right) The amino acid sequence alignment among BoNT/A or BoNT/B subtypes performed using Clustal Omega. Only the VHH-binding epitopes are represented. (PDF) [file ppat.1010169.s007.pdf]

Supplementary Figure 5

A

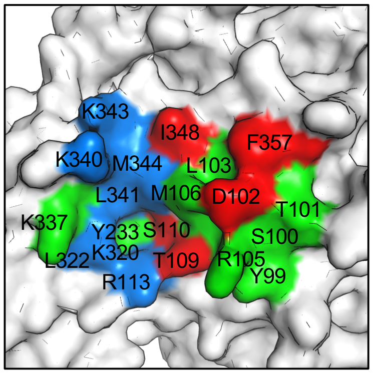

ALc-H7

```
                  1111111112333333333
                 90000000113223444445
                 90123569033027013487
A1               YSTDLRMTSRYKLKKLKMIF
A2               YSTDLRMTSRYKLKKLKMIF
A3               YSTGLRMSFKYKFAEFRVGP
A4               YSTDLRMISRYKLKELKLIF
A5               YSTELRMTSRYKLKKLKMIF
A6               YSTDLRMTSRYKLKKLKMIF
A7               YSTDLRMTSRYKLRKLKMIF
A8               YSTDLRMTSRYKLKKLKMIF
                 *** ***  :**: ::::
```

B

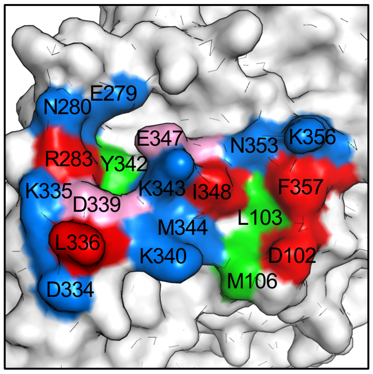

ALc-B8

```
                 1112223333333333333
                 0007883333444444555
                 2369034569023478367
A1               DLMENRDKLDKYKMEINKF
A2               DLMENRDKLDKYKMEINNF
A3               GLMQKSNKAKEYRVRGENP
A4               DLMKKSDRLDEYKLEINKF
A5               ELMENRDKLDKYKMEINKF
A6               DLMENRDKLDKYKMEINKF
A7               DLMENRDKLDKYKMEINKF
A8               DLMENRDKLDKYKMEINKF
                  **:: :: .:*::. ::
```

C

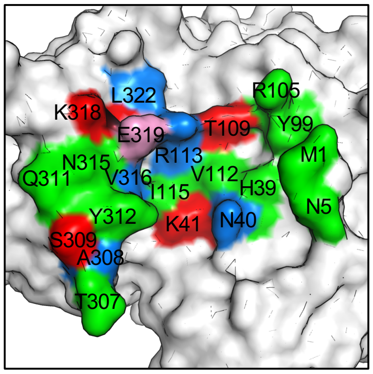

JPU-D12

```
                        11111333333333
                   3449001110001111112
                 159019592357891256892
A1               MNHNKYRTVRITASQYNVKEL
A2               MNHNKYRTVRITASQYNVKEL
A3               MNHEGYRSVKITTPQYNIIRF
A4               MNHNKYRIVRITASQYNVKEL
A5               MNHNKYRTVRITASQYNVKEL
A6               MNHNKYRTVRITASQYNVKEL
A7               MNHNKYRTVRITASQYNVKEL
A8               MNHNKYRTVRITASQYNVKEL
                 ***: ** *:**: ***: .:
```

D

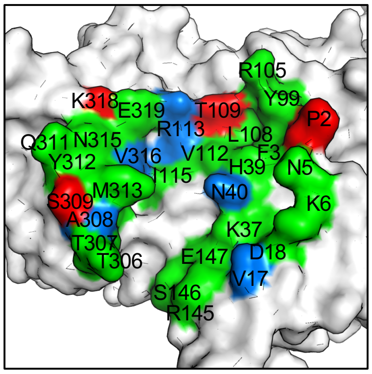

JPU-A11

```
                           11111111133333333333
                     11334900011144400001111111
                 235678790958923556767891235689
A1               PFNKVDKHNYRLTVRIRSETTASQYMNVKE
A2               PFNKVDKHNYRLTVRIRSETTASQYMNVKE
A3               PFNKVDKHEYRLSVKIRSETTTPQYMNIIR
A4               PFNKVDKHNYRLIVRIRSETTASQYMNVKE
A5               LFNKVDKHNYRLTVRIRSETTASQYMNVKE
A6               PFNKVDKHNYRLTVRIRSETTASQYMNVKE
A7               PFNKVDKHNYRLTVRIRSETTASQYMNVKE
A8               PFNKIDKHNYRLTVRIRSETTASQYMNVKE
                  ***:***:*** *:******: ****: .
```

E

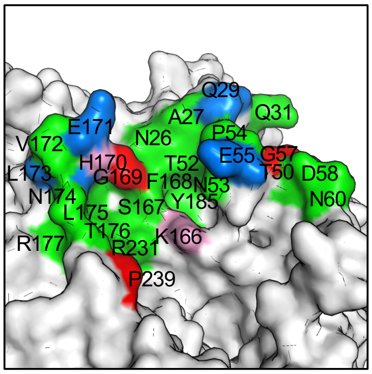

JPU-G3

```
                        111111111111122
            222355555556666677777777833
            679102345780678901234567519
A1          NAQQTTNPEGDNKSFGHEVLNLTRYRP
A2          NAQQTTNPEGDNKSFGHDVLNLTRYRP
A3          NAQQTTNPEGDNKSFGHDVFNLTRYRP
A4          NAKQITNPEVDNSSFRDDVLNLTRYRT
A5          NAQQTTNPEGDNKSFGHDVLNLTRYRP
A6          NAQQTTNPEGDNKSFGHEVLNLTRYRP
A7          NAQQITNPEGDNKSFGHDVLNLTRYRP
A8          NAQQTTNPKGDNKSFGHDVLNLTRYRP
            **:* ***: **.** .:*:******
```

F

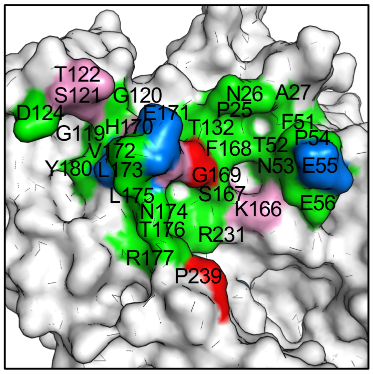

JPU-C1

```
                     111111111111111111122
            222555555122223666677777777833
            567123456901242678901234567019
A1          PNAFTNPEEGGSTDTKSFGHEVLNLTRYRP
A2          PNAFTNPEEGGSTDTKSFGHDVLNLTRYRP
A3          PNAFTNPEEGGSTDTKSFGHDVFNLTRYRP
A4          PNAFTNPEEGGGKDTSSFRDDVLNLTRYRT
A5          PNAFTNPEEGGSTDTKSFGHDVLNLTRYRP
A6          PNAFTNPEEGGSTDTKSFGHEVLNLTRYRP
A7          PNAFTNPEEGGSTDTKSFGHDVLNLTRYRP
A8          PNAFTNPKEGGSTDTKSFGHDVLNLTRYRP
            *******:***..**.** .:*:******
```

G

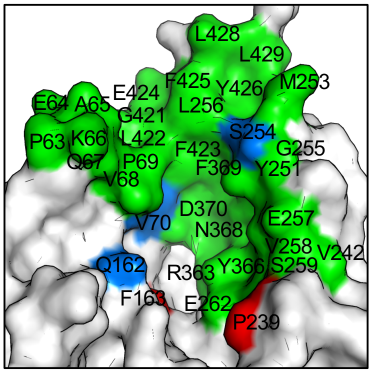

JPU-C10

```
                    11222222222223333344444444
            6666666766345555555566666722222222
            3456789023921345678923689012345689
A1          PEAKQVPVQFPVYMSGLEVSERYNFDGLFEFYLL
A2          PEAKQVPVQFPVYMSGLEVSERYNFDGLFEFYLL
A3          PEAKQVPVQFPVYMSGLEVSERYNFDGLFEFYLL
A4          PEAKQVPIESTVYMAGLEVSERYNFDGLFEFYLL
A5          PEAKQVPVQFPVYMSGLEVSERYNFDGLFEFYLL
A6          PEAKQVPVQFPVYMSGLEVSERYNFDGLFEFYLL
A7          PEAKQVPVNFPVYMSGLEVSERYNFDGLFEFYLL
A8          PEAKQVPVQFPVYMSGLEVSERYNFDGLFEFYLL
            *******::  ***:*******************
```

H

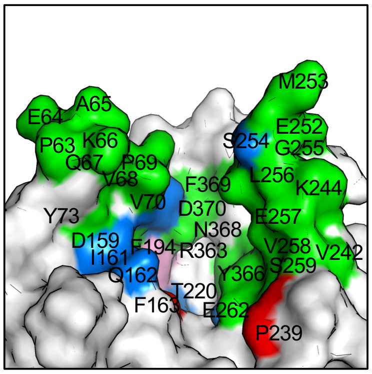

JPU-A5

```
                     1111122222222222223333
            6666666775666923445555555566667
            3456789039123409242345678923690
A1          PEAKQVPVYDIQFFTPVKEMSGLEVSERYFD
A2          PEAKQVPVYDIQFFTPVKEMSGLEVSERYFD
A3          PEAKQVPVYDIQFFTPVKEMSGLEVSERYFD
A4          PEAKQVPIYNIESVATVKEMAGLEVSERYFD
A5          PEAKQVPVYDIQFFTPVKEMSGLEVSERYFD
A6          PEAKQVPVYDIQFFTPVKEMSGLEVSERYFD
A7          PEAKQVPVYDINFFTPVKEMSGLEVSERYFD
A8          PEAKQVPVYDIQFFTPVKEMSGLEVSERYFD
            *******:*:*: .: ****:**********
```

I

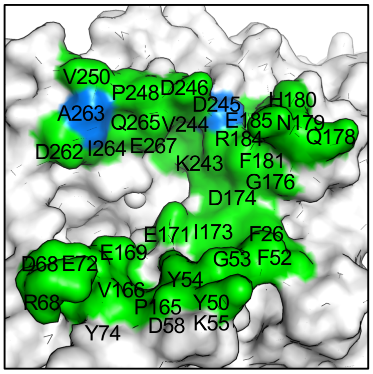


```
JSG-C1
                 111111111111122222222222
          25555556677666777777888844444566666
          60234588924569134689014534568023457
B1        FYFGYKDRDEYPVEEIDGQNHFREKVDDPVDAIQE
B2        FYFGYKDRDEYPVEEIDGQNHFREKVDDPVDAIQE
B3        FYFGYKDRDEYPVEEIDGQNHFREKVDDPVDAIQE
B4        FYFGYKDRDEYPVEEIDGQNHFREKVDDPVDTIQE
B5        FYFGYKDRDEYPVEEIDGQNHFREKVNDPVDAIQE
B6        FYFGYKDRDEYPVEEIDGQNHFREKVDDPVDAIQE
B7        FYFGYKDRDEYPVEEIDGQNHFREKVDDPVDAIQE
B8        FYFGYKDRDEYPVEEIDGQNHFREKVDDPVDAIQE
          **************************:****:***
```

J

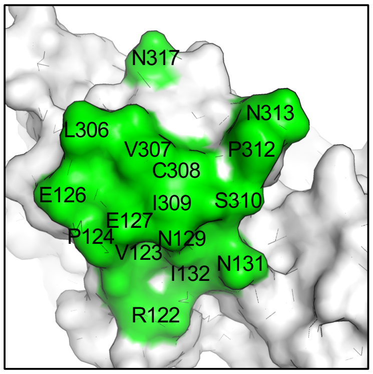


```
JLJ-G3
          1111111133333333
          2222223300001111
          2346791267890237
B1        RVPEENNILVCISPNN
B2        RVPEENNILVCISPNN
B3        RVPEENNILVCISPNN
B4        RVPEENNILVCISPNN
B5        RVPEENNILVCISPNN
B6        RVPEENNILVCISPNN
B7        RVPEENNILVCISPNN
B8        RVPEENNILVCISPNN
          ****************
```
